# ImmunoPET with Zirconium-89 specifically detects postoperative biofilm-associated implant infections: a preclinical study

**DOI:** 10.1186/s13550-026-01421-z

**Published:** 2026-04-08

**Authors:** F. Ruben H. A. Nurmohamed, Kevin J. H. Allen, Connor Frank, Mackenzie E. Malo, J. Fred. F Hooning van Duyvenbode, Berend van der Wildt, Alex J. Poot, Marnix G. E. H. Lam, Jos A. G. van Strijp, H. Charles Vogely, Harrie Weinans, Ekaterina Dadachova, Bart C. H. van der Wal

**Affiliations:** 1 College of Pharmacy and Nutrition, University of Saskatchewan, Saskatoon, Canada; 2 Department of Orthopedics, University Medical Center Utrecht, Utrecht, The Netherlands; 3 Department of Nuclear Medicine, University Medical Center Utrecht, Utrecht, The Netherlands; 4 Department of Medical Microbiology, University Medical Center Utrecht, Utrecht, The Netherlands; 5 Department of Biomechanical Engineering, Delft University of Technology, Delft, The Netherlands; 6 Department of Orthopedics, Leiden University Medical Center, Leiden, The Netherlands

**Keywords:** ImmunoPET, Preclinical infection Imaging, Biofilm-associated Implant infections, Biofilm detection, Postoperative Diagnosis

## Abstract

**Background:**

Early postoperative implant infections are difficult to diagnose due to overlapping symptoms with inflammation. However, prompt surgical intervention for an implant infection can prevent the need for repeated surgeries and improve the overall success of the treatment and preserving the implant. The primary objective of this study was to assess the sensitivity and specificity of a novel immuno-PET radiotracer for detecting *Staphylococcus aureus* bacteria and their biofilms in a preclinical rat model.

**Results:**

An antibody against wall teichoic acid a common surface component of S. aureus, was labeled with Zirconium-89- as the PET tracer. Wistar Han rats underwent surgery with a S. aureus-related biofilm-infected femoral implant on one side and a sterile femoral implant on the contralateral side. The diagnostic efficacy of this imaging modality was compared with clinically established nuclear imaging techniques for implant infections, including [^99m^Tc]Tc-MDP SPECT/CT, [^18^F]FDG PET/CT, and [^18^F]NaF PET/CT. Furthermore, co-injection of unlabeled (“cold”) antibodies was performed to evaluate their impact on biodistribution. All animals with a biofilm-associated femoral implant infection showed significantly higher uptake of the novel ImmunoPET tracer in the infected side compared to the sterile side throughout the 13-day postoperative study duration. A dose-dependent increase in tracer accumulation was observed with co-injection of cold antibody, suggesting its potential to improve biodistribution.

**Conclusions:**

ImmunoPET with Zirconium-89-labeled antibodies specific for wall teichoic acid antigen demonstrates sensitive and specific diagnostic capabilities compared to conventional nuclear imaging modalities, offering a promising tool for early detection of postoperative chronic low-grade infections and septic implant loosening.

**Supplementary Information:**

The online version contains supplementary material available at 10.1186/s13550-026-01421-z.

## Introduction

Timely diagnosis of implant infections is critical to prevent revision surgeries and preserve implants [1, 2]. However, current diagnostic methods such as C-reactive protein (CRP), erythrocyte sedimentation rate (ESR), white blood cell count, and synovial fluid markers, often fail to differentiate infections from postoperative inflammation [3–5].

To enhance diagnostic accuracy, advanced nuclear imaging modalities can be utilized. These include [^99m^Tc]Tc-MDP Single Photon Emission Computed Tomography (SPECT), which employs a bone-seeking radiopharmaceutical that binds to calcium-ions on the bone surface via chemisorption [6]. Furthermore, [^18^F]FDG Positron Emission Tomography (PET) can be used, leveraging a glucose analog as a tracer [7]. Both modalities have been extensively applied and evaluated for diagnosing implant-related infections. However, distinguishing postoperative inflammation from infection remains a significant challenge, underscoring the need for more precise diagnostic tools [8–11].

Additionally, in implant surgery, differentiating between aseptic and septic loosening remains diagnostic challenge [12]. Aseptic loosening involves increased macrophage activity and osteolysis (bone resorption) by osteoclasts due to micro- and nano-scale debris and can be described as an inflammatory process. Likewise, septic loosening caused by bacteria such as *Staphylococcus aureus*, triggers bacterial-induced inflammation and osteolysis [13]. Aseptic loosening is responsible for 29% of early total hip implant failures and 9.2% of total knee implant failures, whereas infections account for 19.5% and 51.3%, respectively [14, 15].

Similar to the challenge of distinguishing between infection and inflammation, conventional diagnostics methods often fail to conclusively differentiate between aseptic and septic prosthetic loosening [16–18]. While [^99m^Tc]Tc-MDP SPECT offers a high sensitivity, it as a low specificity. In contrast, [^18^F]-FDG PET is able to provide satisfactory sensitivity and specificity for detecting an implant infection [16, 19]. However, due to postoperative inflammation (foreign-body reaction), distinguishing between an infection and inflammation within the first three months after surgery remains challenging with [^18^F]-FDG PET analysis [20]. The same challenge applies to fracture-related infections, where [^18^F]FDG PET showing a high false-positive risk post-surgery, highlighting the need for more precise diagnostic tools [21, 22] Diagnosing Fracture-Related-Infection with bone-scintigraphy is also challenging, as it is sensitive but lacks specificity [23].

The application of a specific antibody as carrier for positron-emitting radionuclides represents a novel imaging technique for postoperative detection of an implant- or a fracture-related infection. This molecular imaging modality is named immuno-positron emission tomography (ImmunoPET) [24]. It is an antibody-based imaging that leverages the targeting capability of an antibody to transport positron-emitting radioisotopes for highly sensitive and specific PET imaging [25].

Specific targeting of *Staphylococcus aureus* and its biofilm has been established with an antibody against Wall Teichoic Acid (WTA) glycopolymer [26, 27]. Two studies from our group demonstrated intra-animal specificity of *Staphylococcus aureus* and its biofilm in a subcutaneous infection mouse model with the monoclonal antibody 4497-IgG1 (anti-β-GlcNAc WTA antibody) [28, 29]. To further highlight the potential of this antibody, our recent findings suggest that radioimmunotherapy with the 4497-IgG1 antibody may exert antimicrobial effects against biofilm-associated implant infections, even under leukopenic conditions [30]. Consequently, we hypothesize that ImmunoPET with the targeting precision of the anti-WTA 4497-antibody can specifically and sensitively detect a low-grade infection compared to conventional nuclear imaging modalities in a challenging postoperative setting.

In this preclinical study, we hypothesize that the Zirconium-89-labeled anti-WTA 4497 antibody possesses significant diagnostic potential as a novel ImmunoPET tracer in the early postoperative phase, specifically targeting three-day-matured *Staphylococcus aureus* biofilm infections. Subsequently, the diagnostic performance of the novel tracer was compared with that of [⁹⁹ᵐTc]Tc-MDP SPECT, [¹⁸F]FDG PET, and [¹⁸F]NaF PET, all of which are hypothesized to have limited ability to distinguish between infection and postoperative inflammation in the early phase following implant surgery. Finally, the study investigated the proof-of-principle for co-injecting an excess of unlabeled (cold) 4497 antibody and its potential to favorably modulate biodistribution.

## Materials and methods (condensed)

A concise description of the methods is provided below; a detailed and extended version is available in the Supplementary Materials including radiolabeling, biofilm maturation, surgical procedure, imaging analyses and statistical analysis*.*

### Animal study design

**Fig. 1:**
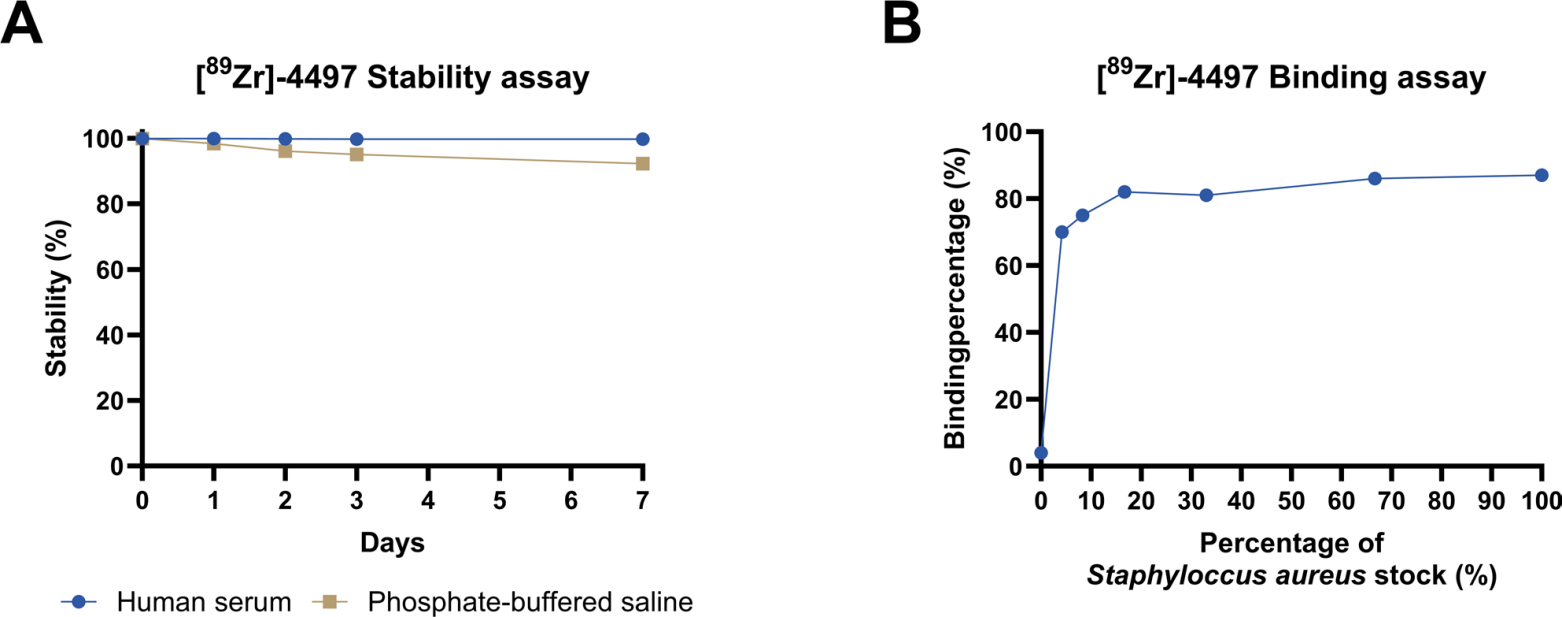
Acceptable stability and binding were achieved with [^89^Zr]−4497 radio-immunoconjugate as ImmunoPET tracer. (**A**) The radiolabeled 4497-antibody was incubated in 90 V/V% human serum or PBS at 37 °C, 3 µl at each time point was withdrawn for iTLC analysis for 7 consecutive days with gamma-counting quantification. The radio-immunoconjugate exhibited excellent stability, with 99.7% stability in human serum and 99.3% in PBS after 7 days. (**B**) At the highest *S. aureus* concentration, the binding affinity was 87% and displayed an immunoreactive fraction of 0.85

This study followed an intra-animal-controlled design. Thirteen male Wistar Han rats, approximately 12–13 weeks old, underwent surgery for the bilateral insertion of intrafemoral implants to distinguish between infected and sterile implants postoperatively. Nine animals received a single injection with 30 µg Zirconium-89-labeled 4497-antibody against the Wall Teichoic Acid glycopolymer as the ImmunoPET tracer ([^89^Zr]Zr-WTA-4497 IgG, hereafter referred to as [^89^Zr]−4497). Of these, three animals additionally received either 300 µg (10×) or 600 µg (20×) of excess unlabeled (cold) 4497 antibody. The in vitro stability test and binding assay can be found in Fig. 1. The in vitro stability assay and in vitro bacterial binding assay of [^89^Zr]−4497 are shown in Fig. 1. The HPLC characterization of [^89^Zr]−4497 conjugated with DFO is shown in Figure S1.

**Fig. 2:**
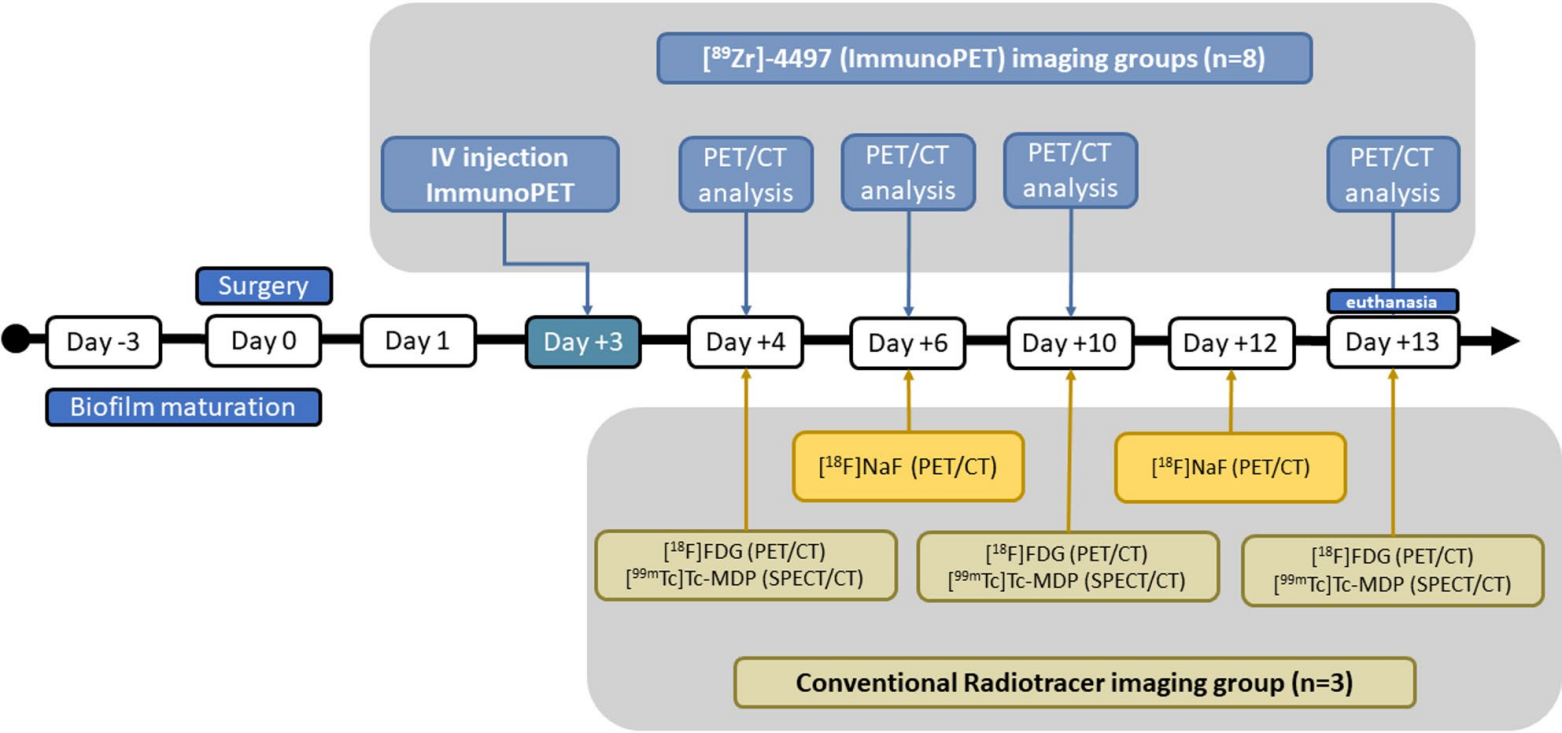
Study timeline of the [^89^Zr]−4497 (ImmunoPET) imaging groups and radiotracer imaging group. After three days of in vitro biofilm maturation on the femoral implants, all animals underwent the bilateral femoral implant procedure on day 0. Subsequently, after three days of infection development, the immunoPET tracer was administered on day + 3. Thereafter, on days + 4, +6, + 10 and + 13 post-surgery, a PET/CT analysis was performed for all animals in the [^89^Zr]−4497 (immunoPET) imaging groups (*n* = 8). Similarly, to the [^89^Zr]−4497 (immunoPET) imaging groups, imaging of the animals receiving the conventional radiotracers (*n* = 3), was performed according to same timeline. All animals from both the immunoPET imaging groups and radiotracer imaging group were euthanized on day + 13 post-surgery for CFU assessment and ex vivo biodistribution assessment (ImmunoPET imaging groups only)

For comparison another four rats received all three conventional radiotracers: [^99m^Tc]Tc-MDP (for bone-scintigraphy SPECT analysis), the glucose analog fluorine-18 FDG (for [^18^F]FDG PET analysis) and the bone tracer fluorine-18 sodium fluoride (for [^18^F]NaF PET analysis) (Fig. 2). One animal from the [^89^Zr]−4497 group with 600 µg co-injection and one animal from the conventional radiotracer group developed an infection of the entire sterile implant side (joint, surrounding bone and implant), and were excluded from the analysis. The ex vivo biodistribution of the excluded animal receiving [^89^Zr]−4497 with 600 µg co-injection after 13 days post-surgery can be found in Fig. S4.

This in vivo experiment was performed after approval of the Animal Research Ethics Board of the University of Saskatchewan, Canada (protocol AUP20230035). All experiments were performed in accordance with institutional guidelines and regulations, and with the ARRIVE guidelines for reporting animal research [31].

### PET/CT and SPECT/CT imaging and data assessment

**Fig. 3:**
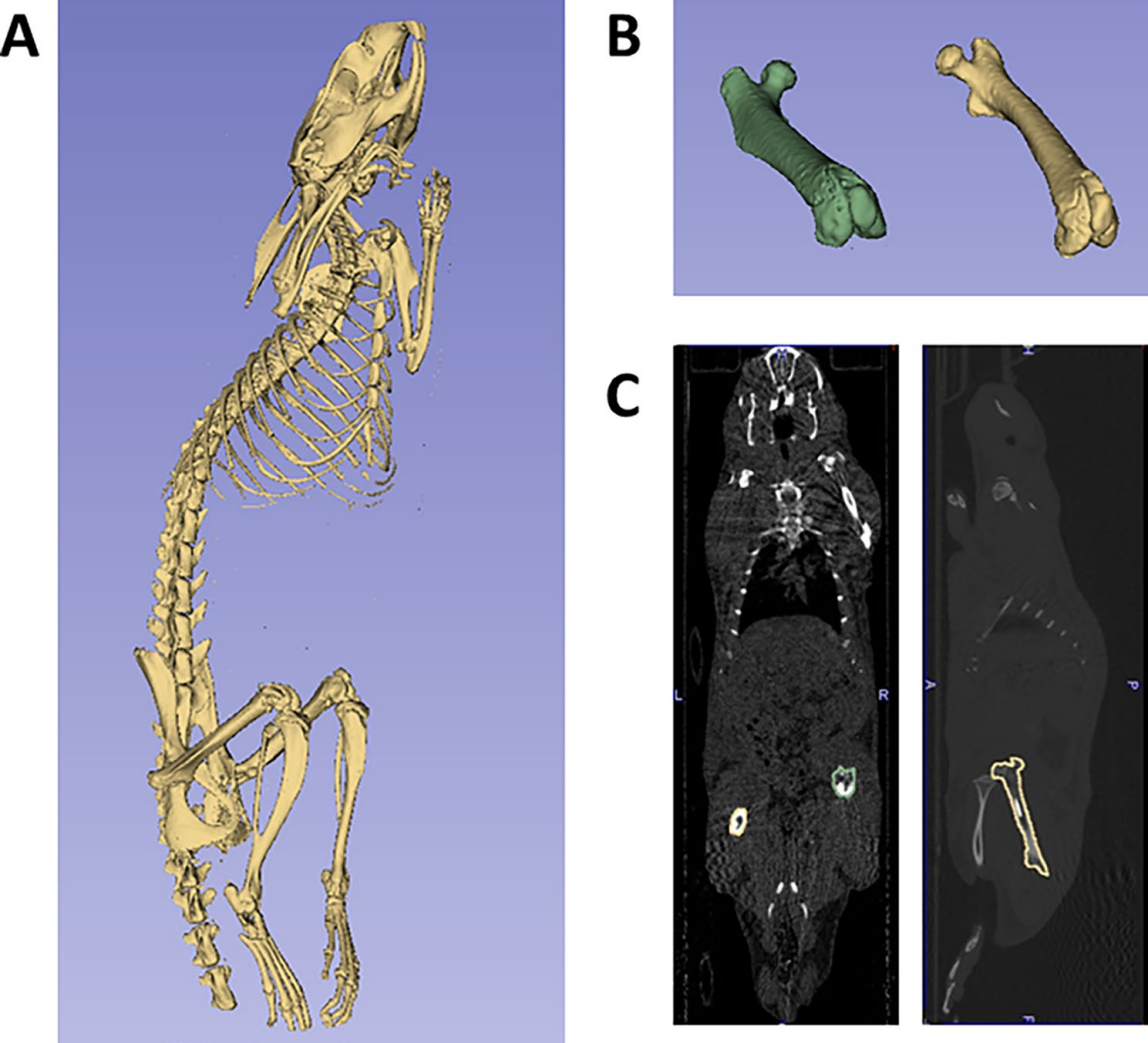
Clear-cut ROIs are used for Standardized Uptake Value analyses. The SUV was normalized to body weight and to the decay of the [^89^Zr]-radioisotope (SUVbw). Calculation of SUVbw was performed by generating specific ROIs. (**a**) A 3D image of the bone was processed from the CT image after manual thresholding. (**b**) The femur with a biofilm-infected implant (green) and the femur with a sterile implant (yellow) were manually isolated. The mask-volume option was used to fill in the gaps in both femurs. (**c**) The posteroanterior and sagittal planes are shown. With clear-cut ROIs, the SUVbw was measured using PMOD software (version 3.910, PMOD Technologies)

Imaging for the ImmunoPET and conventional radiotracer groups was conducted using PET/CT or SPECT/CT with the VECTor^4^CT scanner (MILabs, Netherlands), depending on the radiotracer employed. See Fig. 2 for the study timeline with the postoperative imaging days. To accurately calculate the standardized uptake value per bodyweight (SUVbw) for both the infected and sterile femurs with implants, 3D Slicer v5.6.2 (slicer.org) was used to generate a precisely defined region of interest (ROI) that matched accurately the anatomical structure of the femur [32]. After thresholding the bone from the CT scan, both femoral bones were manually isolated. A Radiotherapy Structure Set (RTSS) DICOM file was created from the 3D image of the femoral bones to generate specific and clear-cut ROIs of the femoral bones. Thereafter, PMOD software (version 3.910, PMOD Technologies) was used to quantify the SUVbw within the ROIs (Fig. 3).

## Results

### Specific infection targeting with [^89^Zr]−4497 and its diagnostic potential

**Fig. 4:**
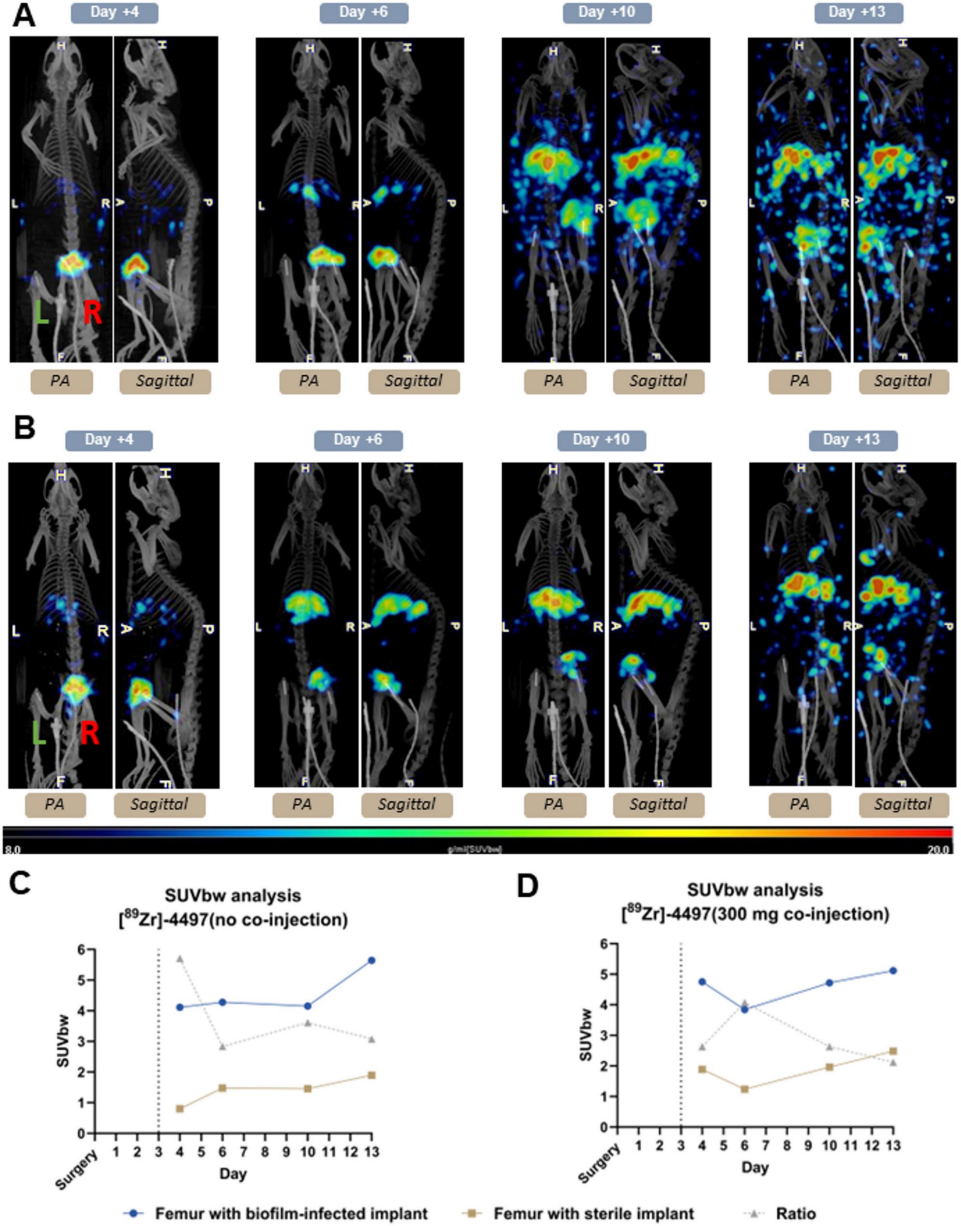
The ImmunoPET tracer shows specific targeting of the infection throughout the study duration. The green L indicates the left sterile implant and the red R indicates the right biofilm-infected implant. Posteroanterior (PA) and sagittal planes are depicted. Dotted line indicates the day of injection. **(a)** PET/CT scans of the [^89^Zr]−4497 with no co-injection of cold 4497-antibody. **(c)** PET/CT scans of [^89^Zr]−4497 (ImmunoPET) with no co-injection show five times more accumulation of the ImmunoPET tracer in the femur with biofilm-infected implant compared to the femur with sterile implant at day four post-surgery. The ratio SUVbw between the femur with biofilm-infected implant and femur with sterile implant on days 4, 6, 10 and 13 post-surgery was 5.71, 2.84, 3.61 and 3.08, respectively. **(b)** PET/CT scans of the [^89^Zr]−4497 with 300 µg co-injection of cold 4497-antibody. **(d)** The ratio SUVbw between the femur with biofilm-infected implant and femur with sterile implant on days 4, 6, 10 and 13 post-surgery was 2.63, 4.07, 2.63 and 2.12, respectively

The PET/CT analyses of the imaging group, [^89^Zr]−4497 with no co-injection of the cold antibody (*n* = 3), showed sensitive and specific bacterial accumulation of the ImmunoPET tracer throughout the study duration (Fig. 4A). The biofilm-infected side (R) exhibited 5.71-, 2.84-, 3.61- and 3.08-fold greater uptake (SUVbw) on days 4, 6, 10 and 13 post-surgery, respectively (Fig. 4C).

**Fig. 5:**
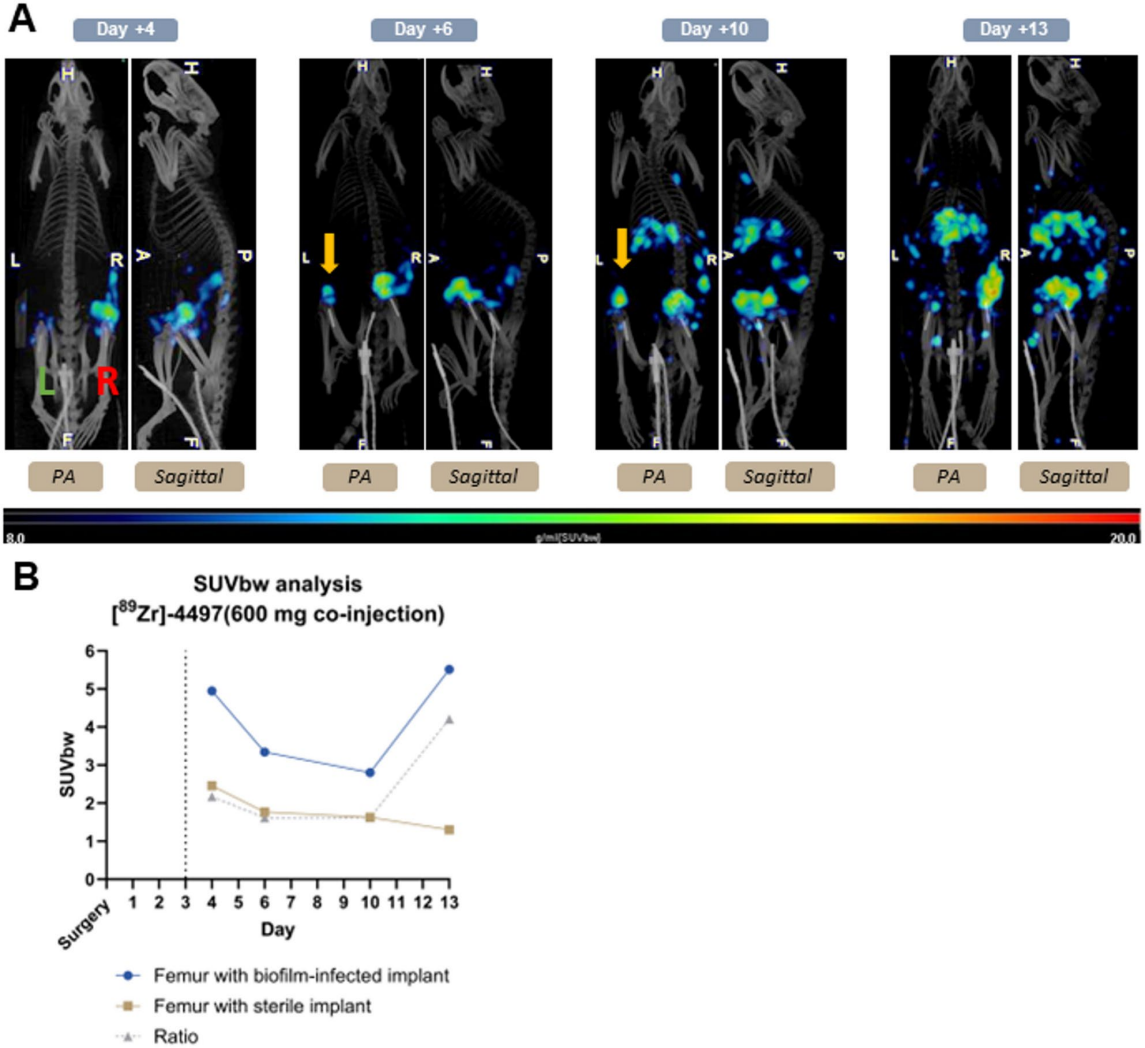
PET/CT scans of [^89^Zr]−4497 (ImmunoPET) co-injected with 600 µg of cold 4497-antibody, demonstrate exceptional sensitivity, revealing accumulation in the contaminated joint on the sterile side. The ImmunoPET tracer shows specific targeting of the biofilm throughout the study duration. The green L indicates the left sterile implant and the red R indicates the right biofilm-infected implant. The yellow arrow displays the left contaminated joint (articular capsule). Posteroanterior (PA) and sagittal planes are depicted. Dotted line indicates the day of injection. **(a)** PET/CT scans of the [^89^Zr]−4497 & 600 µg co-injection of cold 4497-antibody. **(b)** The ratio SUVbw between the femur with biofilm-infected implant and femur with sterile implant on days 4, 6, 10 and 13 post-surgery was 2.17, 1.62, 1.62 and 4.21, respectively

The PET/CT analyses of the imaging groups, [^89^Zr]−4497 with 300 µg (*n* = 3) and 600 µg co-injection (*n* = 2) of the cold 4497-antibody, also showed sensitive and specific accumulation of the ImmunoPET tracer throughout the study duration (Figs. 4B and 5A). The combination with 300 µg of cold antibody exhibited 2.63-, 4.07-, 2.63- and 2.12-fold greater uptake on days 4, 6, 10 and 13 post-surgery in the biofilm-infected side (R), respectively (Fig. 4D). The combination with 600 µg of cold antibody exhibited 2.17-, 1.62-, 1.62- and 4.21-fold greater uptake on days 4, 6, 10 and 13 post-surgery in the biofilm-infected side (R), respectively (Fig. 5B).

A detailed overview of mean SUVbw uptake values across all time points is provided in Supplementary table S3.

Across all ImmunoPET imaging groups (*n* = 8), the mean uptake (SUVbw) of the femur with a biofilm-infected implant (R) was 4.56 ± 0.8, 3.88 ± 1.4, 4.03 ± 1.4 and 5.41 ± 0.8 for postoperative days 4, 6, 10 and 12, respectively. The mean SUVbw of the femur with a sterile implant (L) was much lower with mean uptake values of 1.62 ± 0.9, 1.46 ± 1.0, 1.69 ± 0.6, and 1.97 ± 0.6 for postoperative days 4, 6, 10 and 12, respectively. Thus, a significant difference in uptake was observed between the femur with biofilm-infected implant (R) and the femur with sterile implant (L) on each imaging day, with p-values consistently below 0.001.

### Conventional nuclear imaging techniques lack specificity

**Fig. 6:**
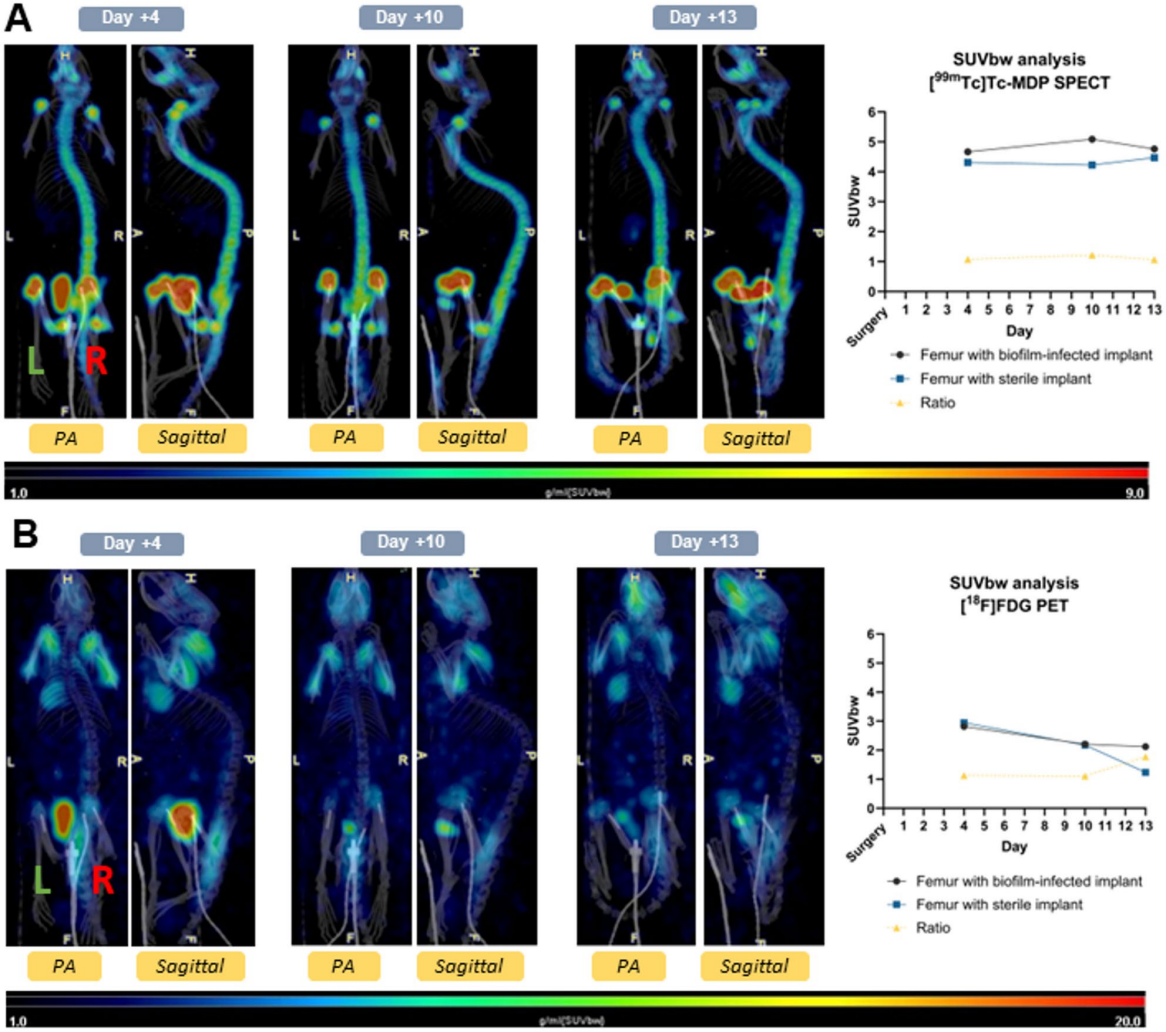
SPECT/CT scans using [^99m^Tc]Tc-MDP (bone scintigraphy) and PET/CT scans with [^18^F]FDG demonstrate comparable uptake at both biofilm-infected and sterile implant sides consistently throughout the study period.Legend: The green L indicates the left sterile implant and the red R indicates the right biofilm-infected implant. The SUVbw and ratio of the femurs with implants are displayed. Posteroanterior (PA) and sagittal planes are depicted. **(a)** [^99m^Tc]Tc-MDP SPECT shows uptake in both implant sites. The ratio SUVbw between femur with biofilm-infected implant and femur with sterile implant on days 4, 10, and 13 post-surgery was 1.07, 1.22, and 1.07, respectively. **(b)** [^18^F]FDG PET shows uptake in both implant sites. The ratio SUVbw between femur with biofilm-infected implant and femur with sterile implant on days 4, 10, and 13 post-surgery was 1.13, 1.10 and, 1.77, respectively

The biofilm-infected side (femur with implant) demonstrated uptake of [^99m^Tc]Tc-MDP comparable to that of the sterile side (femur with implant) with uptake ratios of 1.07, 1.22, and 1.07 on postoperative imaging days 4 (*n* = 3), 10 (*n* = 2) and 13 (*n* = 2), respectively. (Fig. 6A).

The biofilm-infected side (femur with implant) also demonstrated uptake of [^18^F]FDG comparable to that of the sterile side (femur with implant) with uptake ratios of 1.13 and 1.11 on postoperative imaging days 4 (*n* = 3) and 10 (*n* = 2), respectively (Fig. 6B). However, the sterile side demonstrated a decline in [^18^F]FDG uptake after 13 days post-surgery (*n* = 2), resulting in an uptake ratio of 1.78 between the biofilm-infected and sterile sides (Fig. 6B).

**Fig. 7:**
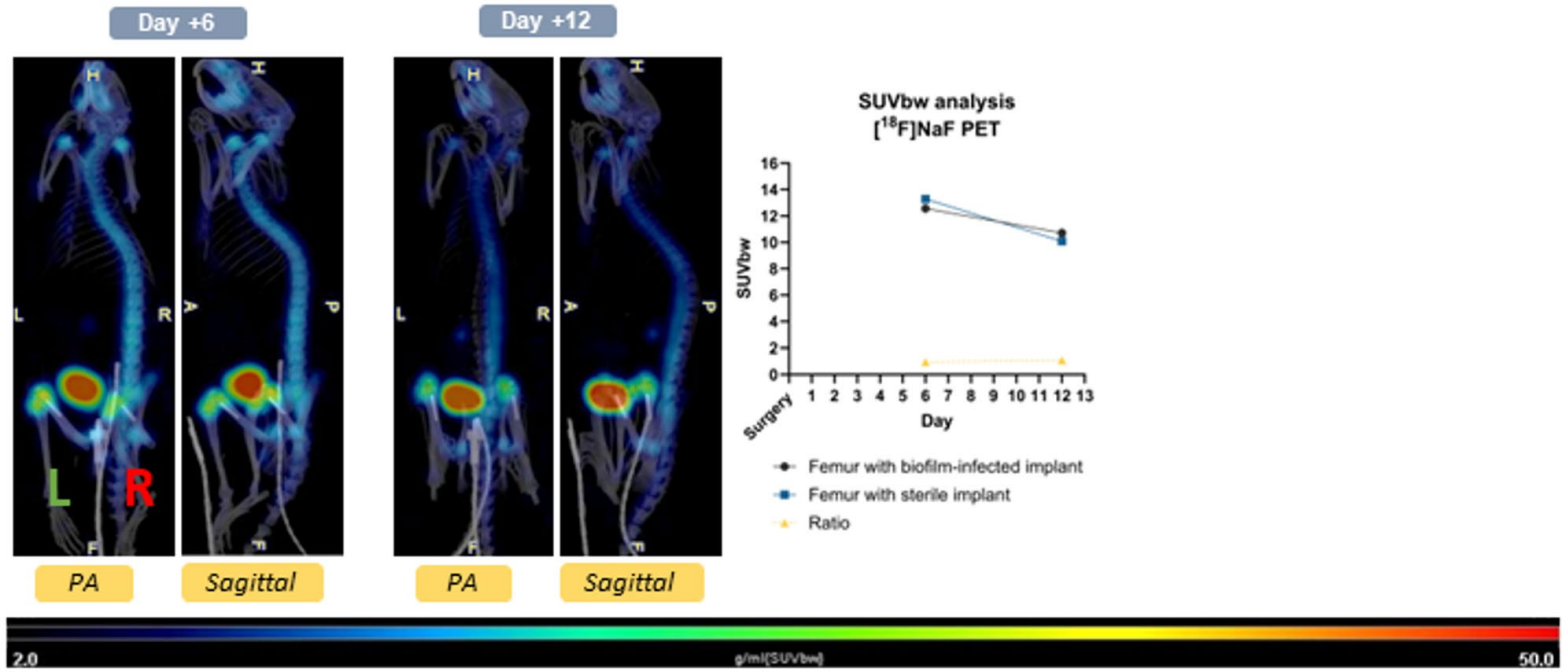
PET/CT scans with [^18^F]NaF demonstrate comparable uptake at both biofilm-infected and sterile implant sites. The green L indicates the left sterile implant and the red R indicates the right biofilm-infected implant. Posteroanterior (PA) and sagittal scans are depicted. The SUVbw and ratio of the femurs with implants are displayed. The ratio SUVbw between the femur with biofilm-infected implant and the femur with sterile implant on days 6 and 12 post-surgery was 0.95 and 1.09, respectively

With [^18^F]NaF PET imaging, the biofilm-infected side (femur with implant) demonstrated uptake of ^18^F-fluoride ions comparable to that of the sterile side (femur with implant) with uptake ratios of 0.95 and 1.09 on postoperative imaging days 6 (*n* = 2) and 12 (*n* = 3) respectively (Fig. 7).

A detailed overview of mean SUVbw uptake values across all time points is provided in Supplementary table S4.

### Ex vivo biodistribution and the proof-of-principle of cold antibody co-injection

**Fig. 8:**
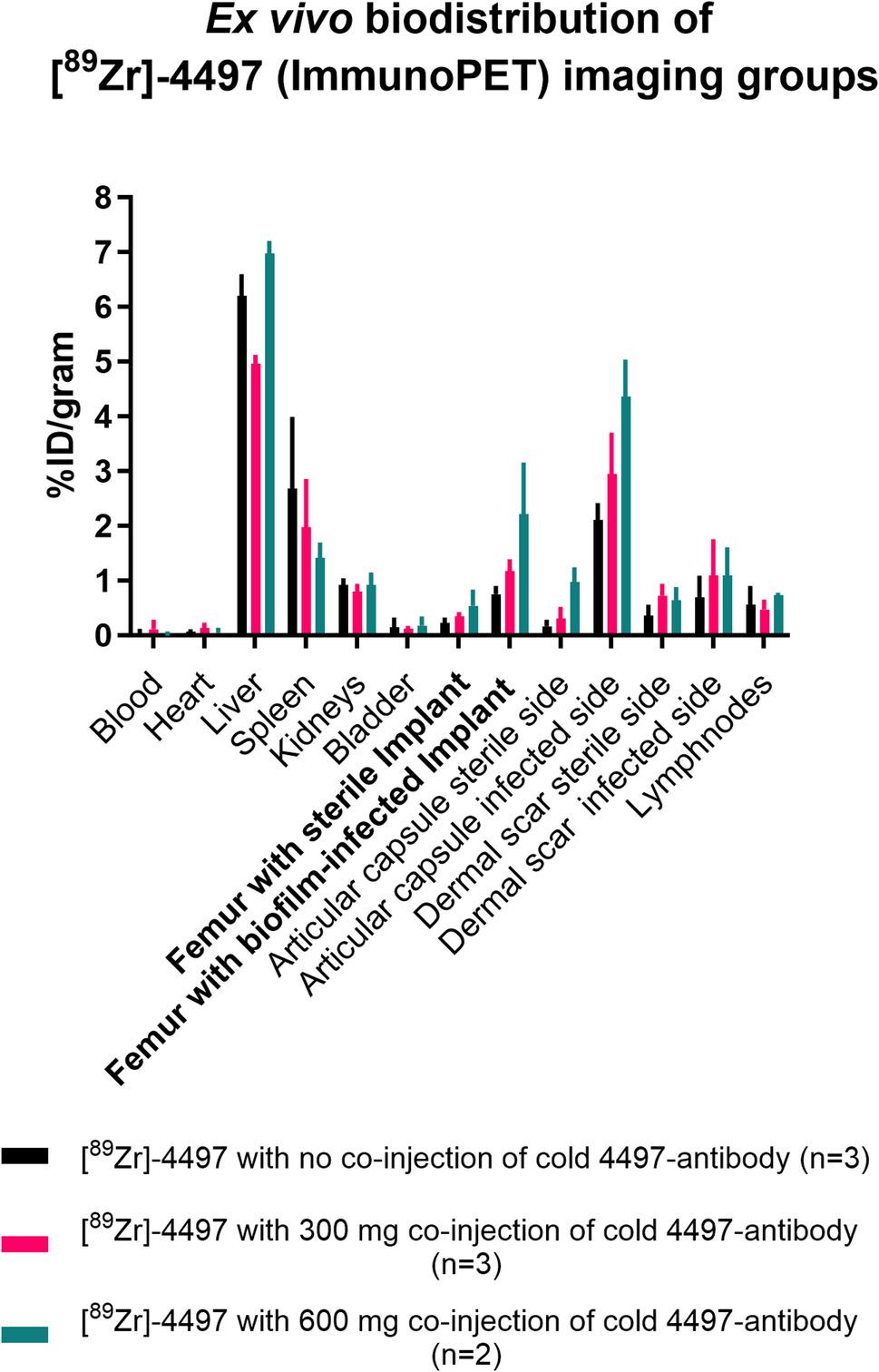
Ex vivo biodistribution of the [89Zr}−4497 ImmunoPET imaging groups demonstrates a dose-dependent increase in radiolabeled antibody accumulation at the biofilm-infected implant site with co-injection. Organs and implants were harvested and individually measured using a gamma counter at 13 days post-surgery. The mean (SD) tissue accumulation of the ImmunoPET tracer Is expressed as %ID/gram. A dose-dependent increase in accumulation is observed in the femur with the biofilm-infected implant and in the articular capsule following the co-injection of cold 4497-antibody. Conversely, a dose-dependent decrease is accumulation in observed in the spleen with co-injection of cold 4497-antibody.

Across all ImmunoPET imaging groups (*n* = 8), all femurs with biofilm-infected implants (R) showed an accumulation of 1.27 ± 0.7%ID/gram. The accumulation of all the femurs with sterile implants (L) was 0.35 ± 0.2%ID/gram. A significant difference was found in the accumulation between the infected side (R) and the sterile side (L) after 13 days post-surgery of the ImmunoPET tracer (*p* = 0.003) (Fig. 8). The ex vivo biodistribution of the excluded [^89^Zr]−4497 animal is shown in Fig. S4.

The liver and spleen also showed considerable uptake with accumulation values of in the range of 5 to 7%ID/gram in the liver and 1.4 to 2.7%ID/gram in the spleen (Fig. 8).

It is hypothesized that a high systemic concentration of the antibody may result in saturation of bacterial binding sites at the most accessible site, which in this study corresponds to the infected joint. Consequently, excess 4497-antibody will preferably bind to the biofilm-infected implant in the femur. This increase in systemically available antibodies is achieved through the administration of a cold-antibody overdose, which also saturates antibody-capturing organs such as the spleen.

In the study, co-injection of the cold 4497-antibody resulted in encouraging effects on the biodistribution between the articular capsule (Fig. S2) at the infected side (R) and the femur with the biofilm-infected implant (R). The ratio of the mean %ID/gram between these two sites from the same infected side, decreased with the co-injection of the cold 4497-antibody. For the no co-injection, 300 µg co-injection, and 600 µg co-injection imaging groups, the calculated %ID/gram ratios between the articular capsule (R) and femur with biofilm-infected implant (R) were 2.8, 2.5, and 1.9, respectively.

Additional results, such as infection/sterility validation (Fig. S3, Tables S1 and S2), ex vivo biodistribution (extended) and short-term hematological effect assessment (Fig. S5) are provided in the Supplementary Results.

**Table 1:** Mechanism of action of Nuclear Agents for Infection and Postoperative Inflammation Imaging in implant-related infections during the first postoperative period

Imaging modality	Mechanism of action	Tracer	Infection	Postoperative inflammation	References
ImmunoPET with [^89^Zr]−4497	Specific binding to the WTA-glycopolymer present on gram-positive bacteria and its biofilm surface.	Anti-β-GlcNAc WTA 4497-antibody	Binding to the WTA-glycopolymer on the bacterial cell wall and surface biofilm.	No binding expected due to the absence of bacteria and biofilm.	[25, 28, 29]
Bone scintigraphy ([^99m^Tc]Tc-MDP SPECT)	MPD Binds to the hydroxyapatite crystals, produced by osteoblasts (chemisorption).	Methyl-diphosphonate (MDP)	MDP uptake is increased by binding to hydroxyapatite crystals formed due to enhanced osteoblastic activity following bacterial internalization.	MDP uptake is increased by binding to hydroxyapatite crystals formed due to enhanced osteoblastic activity following new bone formation.	[33–36]
[^18^F]FDG PET	Activated leucocytes express more GLUT1 and GLUT3 receptors.	Fluorodeoxyglucose (FDG)	FDG uptake is increased in activated leucocytes/macrophages due their response to infection.	FDG uptake in activated leukocytes is increased due to inflammation in tissues, such as during early bone repair.	[20, 37–40]
[^18^F]NaF PET	^18^F-ions exchange with hydroxyl ions of hydroxyapatite crystals which are produced by osteoblasts (chemisorption).	No tracer, ^18^F-NaF will dissociate into Na^+^ and ^18^F-fluoride (F^−^) ions.	^18^F-ions uptake is increased by binding to hydroxyapatite crystals formed due to enhanced osteoblastic activity following bacterial internalization.	^18^F-ions uptake is increased by binding to hydroxyapatite crystals formed due to enhanced osteoblastic activity following new bone formation.	[35, 36], [41–43]

## Discussion

Distinguishing between surgical-related inflammation and an infection in the first postoperative days remains a challenge task with today’s diagnostic tools. In the present study, the potential for diagnostic differentiation between inflammation (represented by a sterile implant, L), and a low-grade infection (represented by a biofilm-infected implant, R), in the first 13 postoperative days was evaluated using the [^89^Zr]-labeled 4497-antibody (as the novel ImmunoPET tracer) and with conventional nuclear imaging modalities such as [^99m^Tc]Tc-MDP-SPECT, [^18^F]FDG-PET, and [^18^F]NaF-PET.

The main issue of implant-associated infections is the presence of biofilm. This biofilm acts as a physical barrier that inhibits full antibiotic penetration and contains diverse types of bacteria such as metabolically inactive bacteria (dormant cells) which are tolerant to antibiotics [44, 45]. Early detection of an implant-associated infection is favorable and could lower the morbidity and mortality [2, 46]. The rationale behind the current novel immunoPET tracer lies in utilizing a highly specific antibody that targets bacteria and their biofilms, which also serves as a carrier for positron-emitting radionuclides (Table 1). In addition, the use of PET imaging is more favorable than SPECT imaging as PET has better image quality and is more suitable for quantification [24]. Hence, we also included [¹⁸F]-NaF in the experiment to image osteoblastic activity, similar to [⁹⁹^m^Tc]-MDP, but using the higher-resolution PET modality. Throughout the complete study duration, significant more uptake (SUVbw) of the novel immunoPET tracer is observed in the femur with the biofilm-infected implant. Even after 13 days post-surgery, significant more accumulation (%ID/gram) was observed in the femur with a biofilm-infected implant, highlighting the selective targeting capabilities of this novel ImmunoPET tracer with the 4497-antibody.

In contrast, due to heightened osteoblastic activity in both the infected and sterile side, increased accumulation of MDP was anticipated (Table 1). Throughout the complete study duration, the SUVbw ratio between the femur with biofilm-infected implant and femur with sterile implant (resembling a postoperative inflammation) showed comparable uptake values with bone scintigraphy ([^99m^Tc]Tc-MDP SPECT). Likewise, due to increased inflammatory processes in both sides, increased glucose (FDG) uptake was anticipated (Table 1). [^18^F]FDG PET analysis on day 4 and 10 post-surgery showed equal glucose uptake. Interestingly, the SUVbw on day 13 post-surgery of the femur with implant from the sterile side showed a decrease in uptake and likely reflects the decreased inflammatory response over the post-surgical time. Similarly, [^18^F]NaF PET runs into the same differentiating issues as bone scintigraphy (Table 1). On day 6 and 12 post-surgery, the uptake value was similar for both sides. In conclusion, conventional nuclear imaging techniques are unable to distinguish between post-surgical inflammation and infection in the early postoperative period, whereas the novel immuno-PET tracer demonstrates the ability to make this distinction.

CFU assessment at the end of the experiment confirmed the presence or absence of infection (Fig. S3). Two animals were excluded from the image analysis due to an infected left (sterile) side. Interestingly, the PET/CT analysis of the [^89^Zr]−4497 (600 µg co-injection) imaging group showed uptake of the ImmunoPET tracer at this left ‘sterile’ side beginning on day 6 post-surgery (Fig. 5). This contamination, could also have resulted from a later acquired infection, received in the cage through bacterial shedding from the animals. As such, utilizing ImmunoPET with PET/CT analysis demonstrated satisfactory sensitivity for an early-stage bacterial focus.

In this study, no significant changes in WBC, RBC, or hemoglobin levels were observed, suggesting that bone marrow suppression did not occur (Fig. S5). Importantly, when using radiolabeled antibodies in subjects with an infection, maintaining the WBC count is crucial, as this is commonly observed in patients receiving radiolabeled antibodies for therapeutic purposes [47]. The thrombocytopenia observed in the present study may be attributed to the surgical intervention in both femoral bones, along with bone marrow infection and *Staphylococcus aureus* bacteremia.

Previous experience with the application of the radiolabeled antibody in in vivo surgical models involving implant infections have demonstrated a complex biodistribution pattern. This complexity arises from the onset of a new infection in the joint, also leading to an arthritis-induced thickened articular capsule (Fig. S2). Inducing an in vivo implant infection may also lead to infection of the wound and joint, resulting in the formation of additional target sites for the immunoPET tracer beyond the femur with the biofilm-infected implant, which are more accessible to the ImmunoPET tracer.

Several animal studies have demonstrated the concept of co-injection of cold antibodies to improve biodistribution [48–51]. Since antibodies transport through convection to inner tissues, organs with loose endothelia (such as the spleen) tend to accumulate antibodies [52]. Building on this principle, the hypothesis behind co-injection with the cold 4497-antibody was that saturating FcR-expressing cells in the spleen would reduce antibody sequestration, thereby increasing systemic availability [53]. The ratio between the infected articular capsule (R) and the femur with biofilm-infected implant (R) decreases with the use of the cold 4497-antibody, suggesting that co-injection indeed enhances systemic availability. However, the use of co-injection should be performed with caution as this could also result in epitope blocking for the radiolabeled 4497-antibody. Leading to a situation where the radiolabeled antibody competes with the unlabeled antibody [54]. A potential effect of competition could be observed in the shift of greatest proportional difference in SUVbw between the femur with biofilm-infected implant and femur with sterile implant. The largest uptake difference between infected side (R) and sterile side (inflamed, L) occurred on day 4 post-surgery in the ImmunoPET group without cold 4497-antibody (ratio of 5.71, Fig. 4C). In contrast, the peak SUVbw ratio shifted to day 6 (4.07, Fig. 4D) and day 13 (4.21, Fig. 5) for the 300 µg and 600 µg co-injection groups, respectively. This shift may result from delayed binding to the biofilm-infected implant due to competition with the co-injected cold-antibody. In a clinical scenario, it is questionable whether adding cold antibodies is necessary. Future animal and human studies should assess the target-to-background ratio and evaluate the need for cold antibody co-injection to enhance delivery of the 4497-antibody.

Another way to strengthen future experimental animal studies is by using a study design that includes both the novel ImmunoPET tracer and [^99m^Tc]Tc-MDP SPECT-radiotracer within the same imaging group. This would eliminate confounding factors such as intra-animal variability, differences in administered doses, and environmental conditions.

The use of radiolabeled antibodies as ImmunoPET tracers for implant-associated infections has been previously explored. Earlier studies have evaluated similar approaches using Zirconium 89 labeled antibodies, including the 1D9-antibody targeting the IsaA antigen and the SAC55-antibody targeting lipoteichoic acid [55, 56]. However, all these experiments lack an in vivo animal model with an intra-animal-controlled design in combination with the complexity of biofilm. Our current study design allows us to evaluate the diagnostic capability in combination with an approach which effectively mimics the characteristics of biofilm-associated infections (a low-grade infection), such as phagocytosis inhibition, chronicity, and low metabolic activity [57–60].

## Conclusion

This biofilm-associated implant infection model, which also resembles a challenging surgical scenario, demonstrated specific and sensitive uptake of the novel ImmunoPET tracer at the infected side throughout the study, performing much better than all the conventional nuclear imaging modalities. Consequently, the introduced ImmunoPET tracer, consisting of Zirconium-89 radiolabeled 4497 antibody targeting wall teichoic acid, represents a highly promising nuclear imaging modality for the diagnosis of a low-grade implant infection in the critical early postoperative period and for the accurate differentiation between aseptic and septic implant loosening. Early differentiation of implant infection from inflammation would enable timely treatment decisions after surgery, potentially leading to improved outcomes in surgical infection treatments. However, before clinical introduction of the proposed ImmunoPET tracer, the versatility of this imaging modality should be further investigated with in vivo studies using various types of pathogens such as e.g. gram-negative bacteria or polymicrobial infections.

## Supplementary Information

Supplementary Material 1

Supplementary Material 2

Supplementary Material 3

Supplementary Material 4

Supplementary Material 5

Supplementary Material 6

## Data Availability

All data are available in the main text or the supplementary materials. Raw data or image files supporting the findings of this study are available from the corresponding author upon reasonable request.

## Abbreviations

[^18^F]FDG: 2-deoxy-2-[¹⁸F]fluoro-D-glucose
[^18^F] Naf: Sodium fluoride-18
4497-antibody: Mut (H + Y) HuIgG1-anti-Wall Techoic Acid-4497 antibody
[^89^Zr]-4497: The 4497-antibody labeled with Zirconium-89
[^99m^Tc]Tc-MDP: Technetium-99 m labeled methylene disphosphonate
CFU: Colony forming units
HE-UHR-RM: High-energy Ultra High-Resolution Rat Mouse
IgG1: Immunoglobulin G subclass 1
ImmunoPET: Immuno-positron emission tomography
OD600: Optical Density at 600 nanometers
PET/CT: Positron Emission Tomography / Computed Tomography
RBC: Red blood cell
SPECT/CT: Single Photon Emission Computed Tomography / Computed Tomography
SUVbw: Standardized Uptake Value (SUV) normalized to body weight (bw)
TSB: Tryptic Soy Broth
WTA: Wall Teichoic Acid
WBC: White Blood Cell
%ID: Percentage Injected Dose

## References

[1] Vrancianu CO, Serban B, Gheorghe-Barbu I, Czobor Barbu I, Cristian RE, Chifiriuc MC, et al. The Challenge of Periprosthetic Joint Infection Diagnosis: From Current Methods to Emerging Biomarkers. Int J Mol Sci. 2023. 10.3390/ijms24054320. 36901750 10.3390/ijms24054320 PMC10002145

[2] Signore A, Sconfienza LM, Borens O, Glaudemans AWJM, Cassar-Pullicino V, Trampuz A, et al. Consensus document for the diagnosis of prosthetic joint infections: a joint paper by the EANM, EBJIS, and ESR (with ESCMID endorsement). Eur J Nucl Med Mol Imaging. 2019;46:971–88. 10.1007/s00259-019-4263-9. 30683987 10.1007/s00259-019-4263-9 PMC6450843

[3] Uvodich ME, Dugdale EM, Osmon DR, Pagnano MW, Berry DJ, Abdel MP. The effectiveness of laboratory tests to predict early postoperative periprosthetic infection after total knee arthroplasty. Bone Joint J. 2021;103:177–84. 10.1302/0301-620X.103B6. 34053291 10.1302/0301-620X.103B6.BJJ-2020-2397.R1

[4] Sukhonthamarn K, Tan TL, Xu C, Kuo FC, Lee MS, Citak M, et al. Determining diagnostic thresholds for acute postoperative periprosthetic joint infection. J Bone Joint Surg. 2020;102:2043–8. 10.2106/JBJS.20.00257. 32941311 10.2106/JBJS.20.00257

[5] Yi PH, Cross MB, Moric M, Sporer SM, Berger RA, Della Valle CJ. The 2013 frank stinchfield award: Diagnosis of infection in the early postoperative period after total hip arthroplasty. Clin Orthop Relat Res. 2014. 10.1007/s11999-013-3089-1. 23884798 10.1007/s11999-013-3089-1 PMC3890203

[6] Adams C, Banks KP. Bone Scan. 2024.

[7] Garg G, Benchekroun MT, Abraham T. FDG-PET/CT in the Postoperative Period: Utility, Expected Findings, Complications, and Pitfalls. Semin Nucl Med. 2017. 10.1053/j.semnuclmed.2017.07.005. 28969758 10.1053/j.semnuclmed.2017.07.005

[8] Govaert GAM, Glaudemans AWJM. Nuclear medicine imaging of posttraumatic osteomyelitis. European Journal of Trauma and Emergency Surgery. 2016. 10.1007/s00068-016-0647-8. 26886235 10.1007/s00068-016-0647-8 PMC4969346

[9] Reuland P, Heiner Winker K, Heuchert T, Ruck P, Muller-Schauenburg W, Weller S, et al. Detection of Infection in Postoperative Orthopedic Patients with Technetium-99m-labeled monoclonal antibodies against Granulocytes. J Nucl Med. 1991;12:2209–14. 1744705

[10] Zhuang H, Sam JW, Chacko TK, Duarte PS, Hickeson M, Feng Q, et al. Rapid normalization of osseous FDG uptake following traumatic or surgical fractures. Eur J Nucl Med Mol Imaging. 2003;30:1096–103. 10.1007/s00259-003-1198-x. 12761597 10.1007/s00259-003-1198-x

[11] Jones-Jackson L, Walker R, Purnell G, McLaren SG, Skinner RA, Thomas JR, et al. Early detection of bone infection and differentiation from post-surgical inflammation using 2-deoxy-2-[18F]-fluoro-D-glucose positron emission tomography (FDG-PET) in an animal model. J Orthop Res. 2005;23:1484–9. 10.1016/j.orthres.2005.03.010.1100230635. 15896941 10.1016/j.orthres.2005.03.010.1100230635

[12] Stumpe KDM, Nötzli HP, Zanetti M, Kamel EM, Hany TF, Görres GW, et al. FDG PET for differentiation of infection and aseptic loosening in total hip replacements: comparison with conventional radiography and three-phase bone scintigraphy. Radiology. 2004;231:333–41. 10.1148/radiol.2312021596. 15044748 10.1148/radiol.2312021596

[13] Hodges NA, Sussman EM, Stegemann JP. Aseptic and septic prosthetic joint loosening: Impact of biomaterial wear on immune cell function, inflammation, and infection. Biomaterials. 2021. 10.1016/j.biomaterials.2021.121127. 34564034 10.1016/j.biomaterials.2021.121127

[14] Melvin JS, Karthikeyan T, Cope R, Fehring TK. Early failures in total hip arthroplasty - a changing paradigm. J Arthroplasty Churchill Livingstone Inc. 2014;29:1285–8. 10.1016/j.arth.2013.12.024. 10.1016/j.arth.2013.12.024 24444568

[15] Postler A, Lützner C, Beyer F, Tille E, Lützner J. Analysis of Total Knee Arthroplasty revision causes. BMC Musculoskelet Disord. 2018. 10.1186/s12891-018-1977-y. 29444666 10.1186/s12891-018-1977-y PMC5813428

[16] Zoccali C, Teori G, Salducca N. The role of FDG-PET in distinguishing between septic and aseptic loosening in hip prosthesis: A review of literature. Int Orthop. 2009. 10.1007/s00264-008-0575-2. 18594820 10.1007/s00264-008-0575-2 PMC2899250

[17] Quinlan ND, Jennings JM. Joint aspiration for diagnosis of chronic periprosthetic joint infection: when, how, and what tests? Arthroplasty. 2023. 10.1186/s42836-023-00199-y. 37658416 10.1186/s42836-023-00199-y PMC10474645

[18] Blanc P, Bonnet E, Giordano G, Monteil J, Salabert AS, Payoux P, et al. The use of labelled leucocyte scintigraphy to evaluate chronic periprosthetic joint infections: a retrospective multicentre study on 168 patients. Eur J Clin Microbiol Infect Dis. 2019;38:1625–31. 10.1007/s10096-019-03587-y. 31218592 10.1007/s10096-019-03587-y PMC6695364

[19] Palestro CJ. Radionuclide imaging of musculoskeletal infection: A review. Journal of Nuclear Medicine. 2016. 10.2967/jnumed.115.157297. 27390160 10.2967/jnumed.115.157297

[20] Kwee RM, Kwee TC, Clin. W.B. Saunders; 2020. 197–205. 10.1016/j.cpet.2019.11.005.

[21] Bosch P, Glaudemans AWJM, de Vries JPPM, Middelberg TR, Govaert GAM, IJpma FFA, et al. Nuclear imaging for diagnosing fracture-related infection. Clin Transl Imaging. 2020;8:289–98. 10.1007/s40336-020-00374-0.

[22] Lemans JVC, Hobbelink MGG, IJpma FFA, Plate JDJ, van den Kieboom J, Bosch P, et al. The diagnostic accuracy of 18 F-FDG PET/CT in diagnosing fracture-related infections. Eur J Nucl Med Mol Imaging. 2019;46:999–1008. 10.1007/s00259-018-4218-6. 10.1007/s00259-018-4218-6 PMC6450834 30523391

[23] He SY, Yu B, Jiang N, Hindawi Limited. Current concepts of fracture-related infection. Int J Clin Pract. 2023. 10.1155/2023/4839701. 37153693 10.1155/2023/4839701 PMC10154639

[24] Wei W, Rosenkrans ZT, Liu J, Huang G, Luo QY, Cai W. ImmunoPET: Concept, Design, and Applications. Chem Rev. 2020. 10.1021/acs.chemrev.9b00738. 32202104 10.1021/acs.chemrev.9b00738 PMC7265988

[25] Knowles SM, Wu AM. Advances in immuno-positron emission tomography: Antibodies for molecular imaging in oncology. Journal of Clinical Oncology. 2012. 10.1200/JCO.2012.42.4887. 22987087 10.1200/JCO.2012.42.4887 PMC3478579

[26] Lehar SM, Pillow T, Xu M, Staben L, Kajihara KK, Vandlen R, et al. Novel antibody-antibiotic conjugate eliminates intracellular *S. aureus*. Nature. 2015;527:323–8. 10.1038/nature16057. 10.1038/nature16057 26536114

[27] Fong R, Kajihara K, Chen M, Hotzel I, Mariathasan S, Hazenbos WLW, et al. Structural investigation of human *S. aureus*-targeting antibodies that bind wall teichoic acid. MAbs. 2018;10:979–91. 10.1080/19420862.2018.1501252. 10.1080/19420862.2018.1501252 PMC6204806 30102105

[28] van Dijk B, Hooning van Duyvenbode JFF, de Vor L, Nurmohamed FRHA, Lam MGEH, Poot AJ, et al. Evaluating the Targeting of a Staphylococcus-aureus-Infected Implant with a Radiolabeled Antibody In Vivo. Int J Mol Sci. 2023. 10.3390/ijms24054374. 36901805 10.3390/ijms24054374 PMC10002501

[29] de Vor L, van Dijk B, van Kessel K, Kavanaugh JS, de Haas C, Aerts PC, et al. Human monoclonal antibodies against Staphylococcus aureus surface antigens recognize in vitro and in vivo biofilm. Elife. 2022. 10.7554/eLife.67301. 34989676 10.7554/eLife.67301 PMC8751199

[30] Nurmohamed FRHA, Allen KJH, Malo ME, Frank C, van Duvenbode JFFH, van der Wildt B, et al. Pathogen-Specific Actinium-225 and Lutetium-177 Labeled Antibodies for Treatment of Biofilm-Associated Implant Infections: Initial In Vivo Proof-of-Concept. Antibiotics. 2025. 10.3390/antibiotics14121283. 41463784 10.3390/antibiotics14121283 PMC12729278

[31] du Sert NP, Ahluwalia A, Alam S, Avey MT, Baker M, Browne WJ, et al. Reporting animal research: Explanation and elaboration for the arrive guidelines 2.0. PLoS Biol. 2020. 10.1371/journal.pbio.3000411. 10.1371/journal.pbio.3000411 PMC7360025 32663221

[32] Fedorov A, Beichel R, Kalpathy-Cramer J, Finet J, Fillion-Robin J-C, Pujol S, et al. 3D slicer as an image computing platform for the Quantitative Imaging Network. Magn Reson Imaging. 2012;30:1323–41. 10.1016/j.mri.2012.05.001. 22770690 10.1016/j.mri.2012.05.001 PMC3466397

[33] Croes M, van der Wal BCH, Vogely HC. Impact of Bacterial Infections on Osteogenesis: Evidence From In Vivo Studies. Journal of Orthopaedic Research. 2019. 10.1002/jor.24422. 31329305 10.1002/jor.24422 PMC6771910

[34] Glaudemans AWJM, Galli F, Pacilio M, Signore A. Leukocyte and bacteria imaging in prosthetic joint infection. Eur Cell Mater AO Res Inst Davos. 2012;25:61–77. 10.22203/ecm.v025a05. 10.22203/ecm.v025a05 23325539

[35] Blake GM, Park-Holohan S-J, Cook GJR, Fogelman I. Quantitative Studies of Bone With the Use of 18F-Fluoride and 99mTc-Methylene Diphosphonate. 2001. 10.1053/snuc.2001.18742 11200203

[36] Komal S, Nadeem S, Faheem Z, Raza A, Sarwer K, Umer H, et al. Localization Mechanisms of Radiopharmaceuticals. Med Isot. 2021. 10.5772/intechopen.94099. IntechOpen.

[37] Glaudemans A, F-FDG PET /. CT in Inflammation and Infection Detection. University Medical Center Groningen.:187–90.

[38] Ting Kung B, Mehdizadeh Seraj S, Zirakchian Zadeh M, Rojulpote C, Kothekar E, Ayubcha C et al. An update on the role of 18 F-FDG-PET/CT in major infectious and inflammatory diseases [Internet]. Am J Nucl Med Mol Imaging. 2019. www.ajnmmi.us/PMC6971480 31976156

[39] Bastian OW, Koenderman L, Alblas J, Leenen LPH, Blokhuis TJ. Neutrophils contribute to fracture healing by synthesizing fibronectin+ extracellular matrix rapidly after injury. Clin Immunol. 2016;164:78–84. 10.1016/j.clim.2016.02.001. 26854617 10.1016/j.clim.2016.02.001

[40] Maimaiti Z, Li Z, Xu C, Fu J, Hao LB, Chen JY, et al. Host Immune Regulation in Implant-Associated Infection (IAI): What Does the Current Evidence Provide Us to Prevent or Treat IAI? Bioengineering. 2023. 10.3390/bioengineering10030356. 36978747 10.3390/bioengineering10030356 PMC10044746

[41] Wilde F, Steinhoff K, Frerich B, Schulz T, Winter K, Hemprich A, et al. Positron-emission tomography imaging in the diagnosis of bisphosphonate-related osteonecrosis of the jaw. Oral Surg Oral Med Oral Pathol Oral Radiol Endod. 2009;107:412–9. 10.1016/j.tripleo.2008.09.019. 19121962 10.1016/j.tripleo.2008.09.019

[42] Czernin J, Satyamurthy N, Schiepers C. Molecular mechanisms of bone 18F-NaF deposition. Journal of Nuclear Medicine. 2010. 10.2967/jnumed.110.077933. 21078790 10.2967/jnumed.110.077933 PMC3169430

[43] Sheppard AJ, Paravastu SS, Wojnowski NM, Osamor CC, Farhadi F, Collins MT, et al. Emerging role of ^18^F-NaF PET/computed tomographic imaging in osteoporosis: a potential upgrade to the osteoporosis toolbox. PET Clin. 2023. 10.1016/j.cpet.2022.09.001. 36442958 10.1016/j.cpet.2022.09.001 PMC9773817

[44] Yin W, Wang Y, Liu L, He J. Biofilms: The microbial “protective clothing” in extreme environments. Int J Mol Sci. 2019. 10.3390/ijms20143423. 31336824 10.3390/ijms20143423 PMC6679078

[45] Wood TK, Knabel SJ, Kwan BW. Bacterial persister cell formation and dormancy. Appl Environ Microbiol. 2013. 10.1128/AEM.02636-13. 24038684 10.1128/AEM.02636-13 PMC3837759

[46] Tripathi S, Tarabichi S, Parvizi J, Rajgopal A. Current relevance of biomarkers in diagnosis of periprosthetic joint infection: an update. Arthroplasty. 2023. 10.1186/s42836-023-00192-5. 37525262 10.1186/s42836-023-00192-5 PMC10391917

[47] Juweid ME, Zhang C-H, Blumenthal RD, Hajjar G, Sharkey RM, Goldenberg DM. Prediction of Hematologic Toxicity After Radioimmunotherapy with I-Labeled Anticarcinoembryonic Antigen Monoclonal Antibodies. J NucI Med. 1999. 10520699

[48] Nedrow JR, Josefsson A, Park S, Ranka S, Roy S, Sgouros G. Imaging of programmed cell death ligand 1: impact of protein concentration on distribution of anti-PD-L1 SPECT agents in an immunocompetent murine model of melanoma. J Nuclear Med Soc Nuclear Med Inc. 2017;58:1560–6. 10.2967/jnumed.117.193268. 10.2967/jnumed.117.193268 PMC5632734 28522738

[49] Van Der Veen EL, Giesen D, Pot-De Jong L, Jorritsma-Smit A, De Vries EGE, Lub-De Hooge MN. 89 Zr-pembrolizumab biodistribution is influenced by PD-1-mediated uptake in lymphoid organs. J Immunother Cancer. 2020. 10.1136/jitc-2020-000938. 33020241 10.1136/jitc-2020-000938 PMC7537332

[50] Josefsson A, Nedrow JR, Park S, Banerjee SR, Rittenbach A, Jammes F, et al. Imaging, biodistribution, and dosimetry of radionuclide-labeled PD-L1 antibody in an immunocompetent mouse model of breast cancer. Cancer Res. 2016;76:472–9. 10.1158/0008-5472.CAN-15-2141. 26554829 10.1158/0008-5472.CAN-15-2141 PMC4715915

[51] Zhao J, Wen X, Li T, Shi S, Xiong C, Wang YA, et al. Concurrent injection of unlabeled antibodies allows positron emission tomography imaging of programmed cell death ligand 1 expression in an orthotopic pancreatic tumor model. ACS Omega. 2020;5:8474–82. 10.1021/acsomega.9b03731. 32337408 10.1021/acsomega.9b03731 PMC7178348

[52] Cataldi M, Vigliotti C, Mosca T, Cammarota MR, Capone D. Emerging role of the spleen in the pharmacokinetics of monoclonal antibodies, nanoparticles and exosomes. Int J Mol Sci MDPI AG. 2017. 10.3390/ijms18061249. 10.3390/ijms18061249 PMC5486072 28604595

[53] Vivier D, Sharma SK, Adumeau P, Rodriguez C, Fung K, Zeglis BM. The impact of FcGRI binding on immuno-PET. J Nuclear Med Soc Nuclear Med Inc. 2019;60:1174–82. 10.2967/jnumed.118.223636. 10.2967/jnumed.118.223636 PMC6681692 30733320

[54] Bouleau A, Nozach H, Dubois S, Kereselidze D, Chevaleyre C, Wang CI, et al. Optimizing immuno-PET imaging of tumor PD-L1 expression: pharmacokinetic, biodistribution, and dosimetric comparisons of 89Zr-labeled anti-PD-L1 antibody formats. J Nucl Med. 2022;63:1259–65. 10.2967/jnumed.121.262967. 10.2967/jnumed.121.262967 PMC9364342 34933891

[55] Zoller SD, Park HY, Olafsen T, Zamilpa C, Burke ZDC, Blumstein G, et al. Multimodal imaging guides surgical management in a preclinical spinal implant infection model. JCI Insight. 2019. 10.1172/jci.insight.124813. 30728332 10.1172/jci.insight.124813 PMC6413782

[56] Pickett JE, Thompson JM, Sadowska A, Tkaczyk C, Sellman BR, Minola A, et al. Molecularly specific detection of bacterial lipoteichoic acid for diagnosis of prosthetic joint infection of the bone. Bone Res. 2018;6:1–8. 10.1038/s41413-018-0014-y. 10.1038/s41413-018-0014-y PMC5916877 29707402

[57] Ciofu O, Moser C, Jensen PØ, Høiby N. Tolerance and resistance of microbial biofilms. Nat Rev Microbiol. 2022. 10.1038/s41579-022-00682-4. 35115704 10.1038/s41579-022-00682-4

[58] Seebach E, Kubatzky KF. Chronic Implant-Related Bone Infections-Can Immune Modulation be a Therapeutic Strategy? Front Immunol. 2019;1724. 10.3389/fimmu.2019.01724. 31396229 10.3389/fimmu.2019.01724 PMC6664079

[59] Gatti M, Barnini S, Guarracino F, Parisio EM, Spinicci M, Viaggi B, et al. Orthopaedic Implant-Associated Staphylococcal Infections: A Critical Reappraisal of Unmet Clinical Needs Associated with the Implementation of the Best Antibiotic Choice. Antibiotics. 2022. 10.3390/antibiotics11030406. 35326869 10.3390/antibiotics11030406 PMC8944676

[60] Crabbé A, Jensen PØ, Bjarnsholt T, Coenye T. Antimicrobial Tolerance and Metabolic Adaptations in Microbial Biofilms. Trends Microbiol. 2019. 10.1016/j.tim.2019.05.003. 31178124 10.1016/j.tim.2019.05.003

