# Supplementary figures and images for: ImmunoPET with Zirconium-89 specifically detects postoperative biofilm-associated implant infections: a preclinical study
Source: EJNMMI Res. 2026 Apr 8;16:79. doi: 10.1186/s13550-026-01421-z (PMC13187111; doi:10.1186/s13550-026-01421-z)

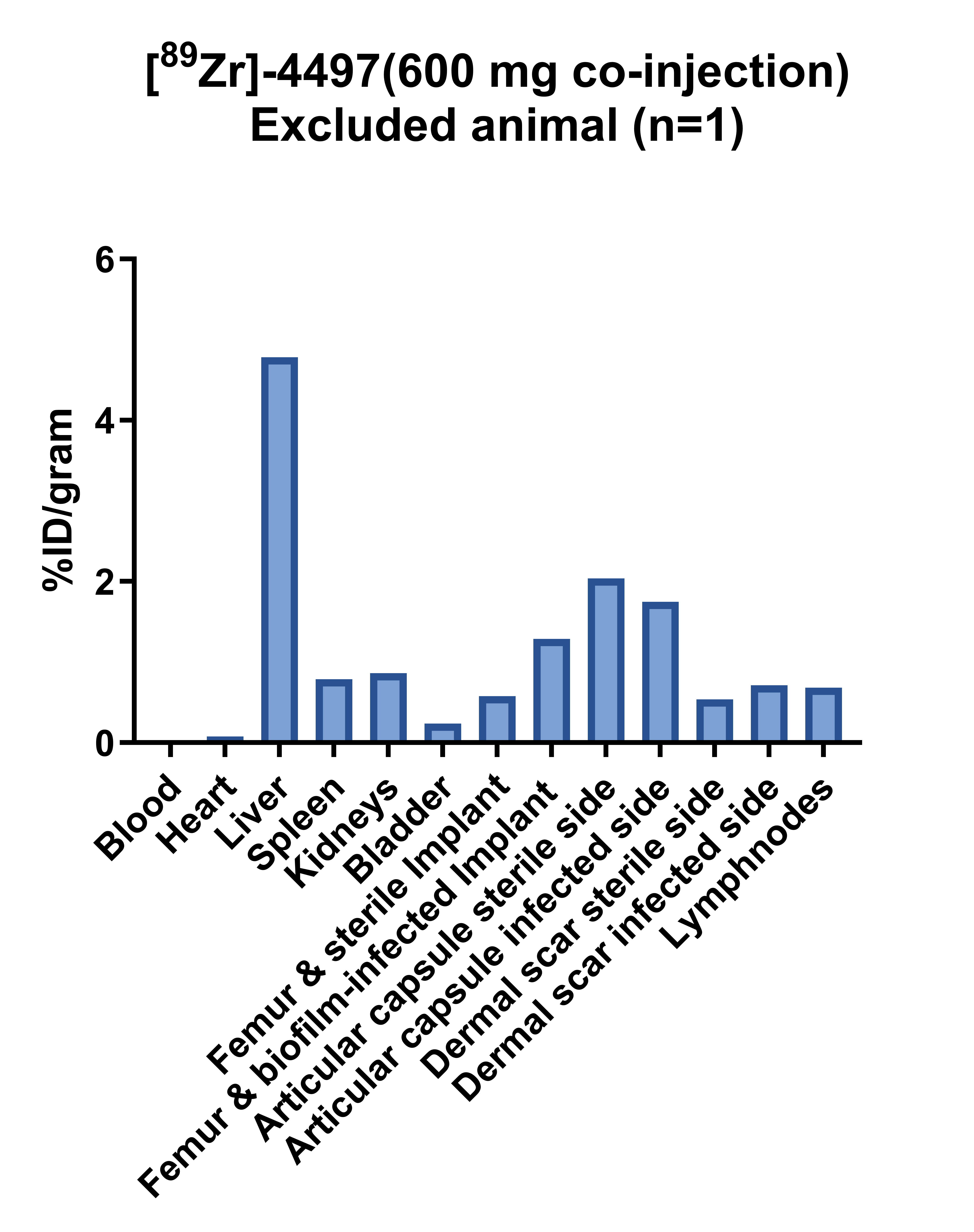

Supplement: Supplementary file 2 — Supplementary Material 2 [file 13550_2026_1421_MOESM2_ESM.tif]

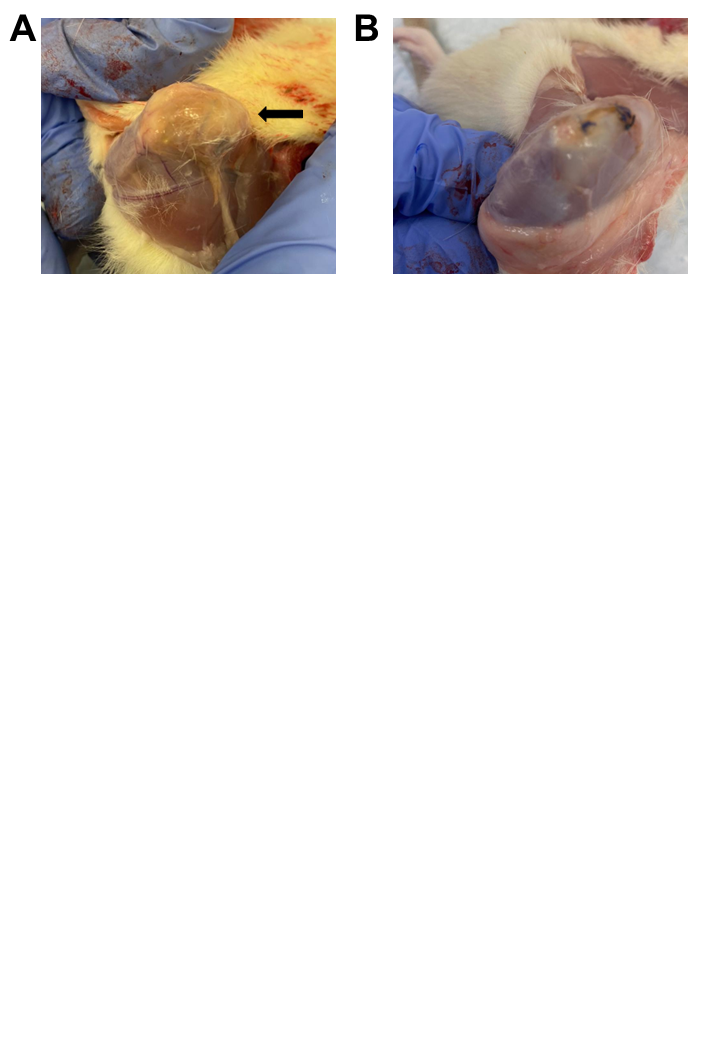

Supplement: Supplementary file 5 — Supplementary Material 5 [file 13550_2026_1421_MOESM5_ESM.tif]

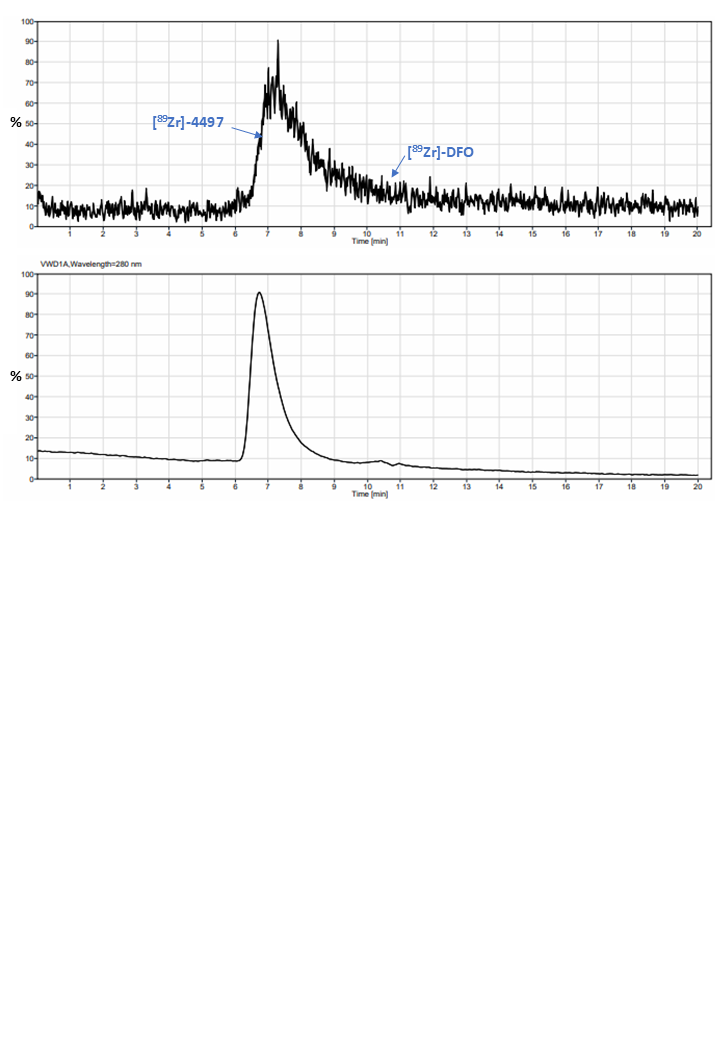

Supplement: Supplementary file 6 — Supplementary Material 6 [file 13550_2026_1421_MOESM6_ESM.tif]
