# Supplementary material for: ImmunoPET with Zirconium-89 specifically detects postoperative biofilm-associated implant infections: a preclinical study
Source: EJNMMI Res. 2026 Apr 8;16:79. doi: 10.1186/s13550-026-01421-z (PMC13187111; doi:10.1186/s13550-026-01421-z)
Supplement: Supplementary file 4 — Supplementary Material 4 [file 13550_2026_1421_MOESM4_ESM.docx]

**Supplemental file**

**ImmunoPET with Zirconium-89 specifically detects postoperative biofilm-associated implant infections. A preclinical study.**

*F. Ruben H.A. Nurmohamed^1,2*^, Kevin J.H. Allen^1^, Connor Frank^1^, Mackenzie E. Malo^1^, J. Fred. F Hooning van Duyvenbode^2^, Berend van der Wildt^3^, Alex J. Poot^3^, Marnix G. E. H. Lam^3^, Jos A. G. van Strijp^4^, H. Charles Vogely^2^, Harrie Weinans^2,5^*^†^*, Ekaterina Dadachova^1^*^†^*and Bart C.H. van der Wal^2^*^†^

*^1^**College of Pharmacy and Nutrition, University of Saskatchewan, Saskatoon, Canada*

*^2^Department of Orthopedics, University Medical Center Utrecht, Utrecht, The Netherlands*

*^3^Department of Nuclear Medicine, University Medical Center Utrecht, Utrecht, The Netherlands*

*^4^Department of Medical Microbiology, University Medical Center Utrecht, Utrecht, The Netherlands*

*^5^Department of Biomechanical Engineering, Delft University of Technology, Delft, The Netherlands*

^†^ *These authors contributed equally to this work*

*First & Corresponding author:

Name Ruben Nurmohamed MD,

Function PhD-candidate and Resident-not-in-training Orthopedic Surgery

Address Burgemeester Reigerstraat 32 bs

3581 KS Utrecht, The Netherlands

Telephone +31610103471

Email: [f.r.h.a.nurmohamed@umcutrecht.nl](mailto:f.r.h.a.nurmohamed@umcutrecht.nl)

[Ruben.nurmohamed@gmail.com](mailto:Ruben.nurmohamed@gmail.com)

ORCHID <https://orcid.org/0000-0001-5369-9853>

**Supplementary methods**

*In vivo* study design

This study followed an intra-animal-controlled design. Thirteen male Wistar Han rats, approximately 12–13 weeks old, underwent surgery for the bilateral insertion of intrafemoral implants to distinguish between infected and sterile implants postoperatively. Nine animals received a single injection with Zirconium-89-labeled 4497-antibody against the Wall Teichoic Acid glycopolymer as the ImmunoPET tracer ([^89^Zr]-4497). For comparison another four rats received all three conventional radiotracers: [^99m^Tc]Tc-MDP (for bone-scintigraphy SPECT analysis), the glucose analog fluorine-18 FDG (for [^18^F]FDG PET analysis) and the bone tracer fluorine-18 sodium fluoride (for [^18^F]NaF PET analysis) (Fig. 1). In addition, 300 µg or 600 µg of the cold 4497-antibody was co-injected with the novel ImmunoPET tracer to potentially improve the biodistribution of the radiolabeled 4497-antibody in this surgical infection model.

The primary study outcome was the measurement and comparison of the Standardized Uptake Value normalized by body weight (SUVbw) of the infected side (femur with a biofilm-infected implant) and the sterile side (femur with a sterile implant). These SUVbw measurements were taken using clear-cut regions of interests (ROIs), following 3D image processing, for both the ImmunoPET and the conventional radiotracer imaging groups.

Secondary outcomes included post-mortem *ex-vivo* biodistribution assessment after co-injection with the two different quantities of the cold 4497-antibody. Additionally, short-term hematological effects were evaluated for all ImmunoPET-receiving rats 13 days post-surgery.

All experiments were performed in accordance with institutional guidelines and regulations, and with the ARRIVE guidelines for reporting animal research [30].

Implant design and Imaging groups

The implants were manufactured with a selective laser melting machine (SLM-125, Realizer, TU Delft, The Netherlands) and were made of medical grade titanium alloy (ELI, Ti6AI4V). To minimize the risk of biofilm delamination during implantation, the implants were engineered with one-millimeter-deep grooves, providing a structured surface for biofilm integration and adherence. These rod-shaped implants were 10 millimeter in length and 0.8 millimeter in diameter.

In total, nine animals received the 4497-antibody radiolabeled with Zirconium-89 (ImmunoPET tracer). In this imaging group, three animals received 30 µg of the ImmunoPET tracer alone, three animals received 30 µg of the ImmunoPET tracer co-injected with 300 µg of the cold antibody (10×), and three animals received 30 µg of the ImmunoPET tracer co-injected with 600 µg of the cold antibody (20×). Four animals received each three different conventional radiotracers ([^99m^Tc]Tc-MDP, [^18^F]FDG and [^18^F]NaF) at different timepoints. All animals were euthanized on day 13 post-surgery using an isoflurane overdose. See Figure 1 for the study timeline with the various imaging groups with the novel and conventional radiotracers.

*In vitro* biofilm maturation

The pathogen *Staphylococcus aureus* USA300 LAC (AH4802) strain was used to grow biofilm on the femoral implants. The bacteria were cultured overnight at 37°C in tryptic soy broth (TSB). The overnight cultured bacteria were re-inoculated into fresh TSB medium and incubated for 3 hours before use, to allow a logarithmic growth.

Before biofilm cultivation on the implant surfaces, all implants were sterilized by autoclaving. These implants were placed in a solution with human fibronectin (Sigma-Aldrich) in carbonate-bicarbonate buffer with a concentration of 20 μg/mL, to facilitate bacterial attachment.

An overnight culture was diluted to an OD_600_ of 1 and then diluted 1:10 in fresh biofilm-medium (TSB containing 0.5% w/v glucose and 3% w/v NaCl). Thereafter, 500 μL was transferred to the implant and incubated statically for three days at 37°C. Each 24 hours, 250 μL of the medium was replaced with fresh biofilm-medium.

To ensure no implantation of planktonic bacteria, the implants with three-days matured biofilm were washed three times with sterile PBS and kept at 4°C until implant surgery.

The 4497-antibody and radiolabeling

In short, the Mut (H+Y) HuIgG1-antiWTA-4497 antibody (the 4497-antibody) was produced by cloning the human constant sections into pcDNA3.4 vectors harboring the variable heavy and light chain sequences *[1]*. These sequences, which were codon optimized and contained KOZAK and HAVT20 signal peptides, were initially taken from B cells of patients infected with *Staphylococcus aureus*. After transfection into EXPI293F cells, the IgG1 antibody was recovered using HiTrap protein A columns from the supernatant in four to five days. Following dialysis in PBS and filter sterilization, the antibodies were examined for aggregation and kept in a concentration of 7.68 mg/mL at 4°C.

Conjugation of the 4497-antibody to the bifunctional chelator p-SCN-Bn-DFO (Macrocyclics, Plano, TX, USA) was performed by exchanging the storage buffer of the antibody with NaHCO_3_ and Na_2_CO_3_ buffer solution that had been passed through a chelex-100 cation exchange resin to remove any advantageous metals (chelexed).*[2]* A 0.5 mL 30k molecular weight cutoff Amicon microconcentrator (Millipore, Burlington, MA, USA) was used to ensure complete exchange of the storage buffer. This process was repeated ten times at 4°C. A three-fold molar excess of DFO was prepared in NaHCO_3_ and Na_2_CO_3_ buffer solution immediately prior to use and subsequently added to the antibody solution. This reaction mixture was incubated at 37°C for 1.5 hour under agitation. The 4497-DFO mixture was then exchanged into a 0.5M HEPES buffer; pH=7.2 (chelexed) by using a 30k molecular weight cutoff Amicon microconcentrator and subsequently centrifuged for ten times at 4°C to remove excess of the p-SCN-Bn-DFO. Upon radiolabeling, ^89^Zr(Ox)_2_ in 1M oxalic acid was dissolved in 0.5M HEPES buffer (chelexed) and neutralized using 1M Na_2_CO_3_. To conclude, 0.37 MBq of Zirconium-89 per µg of 4497-DFO conjugate was used for radiolabeling. The 4497-antibody was then quenched with 0.5mM DTPA to bind any free ^89^Zr. Radiolabeling yield was determined by instant thin-layer chromatography (iTLC) and size exclusion high pressure liquid chromatography (SEC HPLC). For iTLC a 1 ul spot was placed on silica gel iTLC stip cut to 1 cm by 8 cm (Agilent Technologies Inc., Santa Clara, CA, USA) with 0.5 mM EDTA as the mobile phase. The strip was cut in half and measured using a gamma counter (2470 Wizard2 Gamma counter, PerkinElmer, Waltham, MA, USA). The bottom half retains the antibody and the top half contains ^89^ZrDTPA. The sample was also run on an Agilent HPLC (Agilent Technologies Inc., Santa Clara, CA, USA) equipped with a UV detector monitoring at UV=280, a BioScan radioactivity detector (Eckert & Ziegler, Valencia, CA, USA), and a TSKgel SuperSw2000, 4.6mm I.D. X 30 cm, 4µm SEC column (TOSOH Bioscience, Tokyo, Japan). An isocratic method was run for 20 minutes with a 150mM Sodium Phosphate buffer, pH 7, as the eluant with [^89^Zr]-4497 showing up at 6.8 min and [89Zr]-DTPA, if present, showing up at 10.8 min. The HPLC characterization of [^89^Zr]-4497 conjugated with DFO is shown in Figure S1.

In vitro binding assay:

The binding assay was performed using seven 1.5 mL microcentrifuge tubes pre-blocked with 2% bovine serum albumin (BSA) in phosphate-buffered saline (PBS) for 1 h to minimize nonspecific binding to tube surfaces. Then, seven serial dilutions of a *S. aureus* suspension were then prepared in 2.0% BSA in PBS. Subsequently, 40 ng/mL of [^89^Zr]-4497 was added to each sample. Samples were incubated at 37°C for 60 min on a thermomixer set to 400 rpm, followed by centrifugation at 17,000 × g for 4 min. The supernatants were transferred to fresh tubes, and the pellets were washed three times with PBS. Radioactivity in both pellets and supernatants was measured using a gamma counter to determine the percentage of [^89^Zr]-4497 bound to planktonic *S. aureus* and the percentage of unbound [^89^Zr]-4497. The cell-binding fraction was calculated as the ratio of radioactivity in the pellet to the total sample radioactivity. A double-inverse plot of total radioactivity versus the inverse ratio of cell-bound activity to cell concentration was generated, and a linear regression was fitted to the data. Immune reactive fraction was determined according to the method described by Lindmo et al., where the reciprocal of the y-intercept represents the immunoreactive fraction under conditions of infinite antigen excess [3].

Bilateral intrafemoral implant procedure

Both femoral implants were inserted in the same surgery. The sterile implant was always inserted into the intramedullary canal of the left femur, while the biofilm-infected implant was always inserted into the intramedullary canal of the right femur. To avoid contamination, the sterile implant was inserted first, and the wound was closed before inserting the biofilm-infected implant in the contralateral leg.

All animals were placed onto a heat blanket and anesthetized with isoflurane (induction dose 5% and maintaining dose of 2-3%) and 1 L/min oxygen. Prior to the start of the surgery, 2 mL NaCl 0.9%, slow-release buprenorphine (0.05 mg/kg) and meloxicam (2 mg/kg) were administered subcutaneously. Ophthalmic ointment was applied to both eyes to prevent corneal drying. After sufficient level of sedation was reached, lidocaine-hydrochloride (7 mg/kg) was locally administered to the procedure site. Preoperative preparation included disinfecting the left leg with Softasept® N (B. Braun) twice, followed by the placement of a one-hole surgical drape around the leg. A midline incision was made on the left knee and the patella was displaced medially exposing the femoral head. An insertion hole was drilled between the two femoral condyles using a hand drill with a length of 1 cm and a width of 1 mm. The sterile implant was subsequently inserted and the opening was closed with bone wax. Absorbable sutures were used to reposition the patella and close the inner tissues. Non-absorbable sutures were used to close the skin. The same procedure was performed for the biofilm-infected implant in the right intramedullary canal. Surgical gloves were changed between legs. Finally, 2 mL NaCl 0.9% was administered once more.

Postoperative management consisted of slow-release buprenorphine (0.05 mg/kg) every other day until 7 days postoperative and meloxicam (2 mg/kg) every day until 3 days postoperative. DietGel® Recovery (ClearH2O®) and DietGel® Boost (ClearH2O®) were given each day throughout the study duration to maintain body weight after surgery.

Administration and imaging analyses

The Sylvia Fedoruk Canadian Centre for Nuclear Innovation provided clinical grade [^99m^Tc]Tc-MDP, [^18^F]FDG, and [^18^F]NaF. Prior to all injections, the animals were weighed and placed into a rat restrainer. All imaging interventions were administered intravenously via the tail. See Fig. 1 for the study timeline with the postoperative imaging days.

Due to slight variations in injection precision, there were minor differences in the injected dose among the ImmunoPET and conventional radiotracer imaging groups. For ImmunoPET analyses, [^89^Zr]-4497 was administered as a single injection, three days following the bilateral intrafemoral implant procedure. The mean injected dose per animal was 9.00 ± 0.22 MBq, 10.85 ± 0.24 MBq and 10.89 ± 0.13 MBq for the [^89^Zr]-4497(no co-injection), [^89^Zr]-4497(300 µg co-injection) and [^89^Zr]-4497(600 µg co-injection) imaging group, respectively.

Using bone scintigraphy ([^99m^Tc]Tc-MDP SPECT), the phosphonate groups of methyl diphosphonate (MDP) interact with calcium ions found in hydroxyapatite crystals formed by osteoblasts (Table 1). For bone-scintigraphy analysis, [^99m^Tc]Tc-MDP was administered on each designated imaging day (Fig. 1). Subsequently, a SPECT/CT scan was performed three hours post-injection. The mean injected dose was 19.1 ± 0.59 MBq, 19.7 ± 0.15 MBq and 14.4 ± 7.90 MBq of [^99m^Tc]Tc-MDP for postoperative imaging day 4, 10 and 13, respectively.

Using the glucose analog 2-deoxy-2-[^18^F]-d-glucose ([^18^F]FDG), uptake is facilitated by increased upregulation of GLUT receptors by inflammatory cells (Table 1). For [^18^F]FDG PET analysis, [^18^F]FDG was administered on each designated imaging day (Fig. 1). Subsequently, a PET/CT scan was performed three hours post-injection. Prior to the day of imaging, all animals receiving [^18^F]FDG, fasted for 18 hours (Fig. 1). Water was ad libitum. The mean injected dose was 22.6 ± 2.41 MBq, 20.3 ± 0.59 MBq and 16.4 ± 6.07 MBq of [^18^F]FDG for postoperative imaging day 4, 10 and 13, respectively.

After endogenous dissociation of [^18^F]NaF into sodium ions and ^18^F-fluoride ions. The ^18^F-fluoride ions are incorporated into the newly formed hydroxyapatite crystals by replacing the hydroxide ions in the hydroxyapatite crystals, particularly when bone turnover is increased (Table 1). This is demonstrated in the following reaction:

Ca_10_(PO_4_)_6_(OH)_2_+2**F**^−^→Ca_10_(PO_4_)_6_**F**_2_+2OH^−^

For [^18^F]NaF analysis, [^18^F]NaF was administered on each designated imaging day (Fig. 1). Subsequently, a PET/CT scan was performed one-hour post-injection. The mean injected dose was 21.3 ± 0.48 MBq and 18.2 ± 1.91 MBq of [^18^F]NaF for postoperative imaging day 6 and 12, respectively.

Imaging analyses

All animals undergoing imaging received 2.5 mL NaCl 0.9% subcutaneously before anesthesia. Eye ointment was subsequently applied. Anesthesia was induced with isoflurane at a 5% induction dose and 2% maintaining dose (1 L/min oxygen). Body temperature was monitored throughout the scan duration. All PET/CT and SPECT/CT scans were performed with the VECTor^4^CT scanner (MILabs).

All animals receiving the [^89^Zr]-labeled antibody underwent a 20-minute PET scan specific for Zirconium-89, utilizing energy windows of 511 keV and 908 keV with a background weight of 4. Subsequently, PET/CT scans were conducted on days 4, 6, 10, and 12 post-surgery.

The [^99m^Tc]Tc-MDP and [^18^F]FDG were co-injected, and after the appropriate uptake period, a simultaneous SPECT and PET scan was performed with a scan duration of 20 minutes. The energy window for [^99m^Tc]Tc-MDP SPECT was set at 140 keV, while for [^18^F]FDG PET, it was set at 511 keV. The **[^18^F]NaF** was administered separately, and the energy window for **[^18^F]NaF PET** analysis was set at **511 keV**. The HE-UHR-RM (high-energy Ultra High-Resolution Rat Mouse) collimator was used for the PET/SPECT/CT analysis in the conventional radiotracer group. For attenuation correction and anatomic reference, a CT scan (55 kV, 0.37 mA) was performed prior to all PET and SPECT scans. A pixel-based algorithm with 16 subsets, 10 iterations and a 0.12 mm^2^ voxel size was used for reconstruction of the PET/CT and SPECT/CT analyses with the MIlabs Reconstruction 8.00 RC6 software (MIlabs).

PET/CT and SPECT/CT data assessment

After imaging, all animals were weighed for normalization of the Standardized Uptake Value by body weight calculation (SUVbw). All SPECT/CT and PET/CT scans were registered to their corresponding CT scan and attenuation corrected. Subsequently, quantification of all scans was performed. To accurately calculate the SUVbw for both the infected and sterile femurs with implants, 3D Slicer v5.6.2 (slicer.org) was used to generate a precisely defined ROI that matched accurately the anatomical structure of the femur *[4]*. After thresholding the bone from the CT scan, both femoral bones were manually isolated. A RTSS DICOM file was created from the 3D image of the femoral bones to generate specific and clear-cut ROIs of the femoral bones. Thereafter, PMOD software (version 3.910, PMOD Technologies) was used to quantify the SUVbw within the ROIs (Fig. 2).

Postmortem sterility and infection assessment

Before biofilm maturation, three randomly selected autoclaved implants and the biofilm growth medium were tested for bacterial growth to confirm the absence of any pathogens.

The femur with implant and the articular capsule (Fig. S2) from both the infected and sterile side were obtained on the 13^th^ day post bilateral intrafemoral implant procedure. The femoral bones with implant were rinsed with sterile PBS and stored in ice throughout the assessment. Aseptically, the implant was separated from the femur and stored in 1 mL sterile PBS. The femur and articular capsule were individually pulverized and homogenized in 20 mL sterile PBS with a Kinematica® Polytron® (PT 10). After 10 minutes of sonication, 10-fold serial dilutions were cultured from the PBS on Colombia Blood Agar plates in duplicates. The CFU per mL were counted after overnight incubation in a 37°C incubator.

*Ex vivo* biodistribution assessment

Organs and both femoral bones were obtained on the 13^th^ postoperative day to assess biodistribution of the ImmunoPET tracer for all [^89^Zr]-4497 (ImmunoPET) imaging groups. The samples were weighed, and accumulated gamma emission was measured with a gamma counter (Perkin Elmer). A standard was created with 10% of the injected dose for each imaging group, which was used to define the counts corresponding to 100% of the injected dose value. This standard was kept in 0.15M ammonium acetate throughout the study duration and was counted together with the dissected organs and femoral bones with implants. All accumulated radioactivity was reported in %ID/g (percentage injected dose per gram). This was calculated by the following formula:

$$\% Injected dose per gram \left( \%\frac{ID}{gram} \right)=\frac{Counts per minute \left( \mathrm{CPM} \right)}{Organ weight \left( \mathrm{gram} \right) \times\left( 10 \times CPM standard \right)}\times100\%$$

Short-term hematological effect assessment

Heart puncture was performed immediately after termination to collect blood for short-term hematological effect assessment. Blood was stored in Lithium Heparin tubes (BD Microtainer). Immediately thereafter, white blood cell count (WBC), red blood cell count (RBC), Hemoglobin level, and Platelet count were assessed using a Beckman-Coulter Ac∙T differential hematology analyzer.

Statistical analysis

Graph presentation and statistical analysis were performed using GraphPad Prism 10. Colony-forming units per mL (CFU/mL) were reported with mean and range. Standardized Uptake Value was normalized to body weight and decay (SUVbw). The mean and ratio (SUVbw femur with biofilm-infected implant / SUVbw femur with sterile implant) were reported. One-tailed paired t-test was used to assess significant differences in SUVbw and %ID/gram between the femur with biofilm-infected implant and femur with sterile implant. All reported hematological parameters were compared to reference clinical lab parameters for male Wistar Han rats provided by Charles River *[5]*.

**Supplementary results**

Preclinical infection model validation

Bacterial assessment of the used biofilm medium for the biofilm growth showed no detectable colonies, ensuring no introduction of pathogens other than the *Staphylococcus aureus* AH4802 bacteria for the biofilm maturation process. Bacterial assessment of the autoclaved sterile implants also showed no detectable colonies.

Adequate infection induction with three-day matured biofilm was achieved for all groups (Fig. S3A-C). The bacterial counts of the sterile implant and surrounding bone were below detectable limits for all animals included in the [^89^Zr]-4497 imaging groups and conventional radiotracer group. However, three animals, one from the [^89^Zr]-4497 with 300 µg co-injection, one from ^89^Zr-4497 with 600 µg co-injection group and one from the conventional radiotracer group developed an infected joint on sterile side resulting in an infected articular capsule after 13 days (Fig. S3B-D).

One animal from the [^89^Zr]-4497 group with 600 µg co-injection and one animal from the conventional radiotracer group developed an infection of the entire sterile implant side (joint, surrounding bone and implant), and were excluded from the analysis. The *ex vivo* biodistribution of the excluded animal receiving [^89^Zr]-4497 with 600 µg co-injection after 13 days post-surgery can be found in Fig. S4.

Overall, a well-developed bacterial infection on the implant, in the surrounding bone, and in the joint (articular capsule) was confirmed on the infected side. On the sterile contralateral side, a concurrent bacterial infection was observed in the joint (articular capsule) in five rats. Importantly, all included animals had a clean femur on the side with the sterile implant. No animal showed impaired mobility after the bilateral femoral implant procedure. Bacterial counts (CFU/mL) for all individual animals including the excluded animals, can be found in Supplementary table S1 and S2.

*Ex vivo* biodistribution of [^89^Zr]-4497 shows significant difference in accumulation

The biodistribution of the [^89^Zr]-4497(no co-injection) imaging group (n=3) demonstrated that the femur with the biofilm‐infected implant (R) exhibited three times greater accumulation than the femur with the sterile implant (L). The measured values were 0.75 ± 0.1 %ID/gram (R) and 0.23 ± 0.1 %ID/gram (L). The articular capsule on the infected side (R) exhibited 13 times greater accumulation compared to that on the sterile side (L). The results were 2.11 ± 0.3 %ID/gram and 0.165 ± 0.1 %ID/gram, respectively.

Using 300 µg or 600 µg co-injections with “cold” 4497 antibody further underscore the sensitivity and specificity of the immunoPET prove with accumulation ratios of 3 and 4 between the infected (R) and the sterile (L) femur with implant, respectively. The articular capsule on the infected side (R) exhibited 9- and 7-times greater accumulation compared to that on the sterile side (L) for the [^89^Zr]-4497(300 µg co-injection) and [^89^Zr]-4497(600 µg co-injection) imaging groups, respectively.

Across all ImmunoPET imaging groups (n=8), all femurs with biofilm-infected implants (R) showed an accumulation of 1.27 ± 0.7 %ID/gram. The accumulation of all the femurs with sterile implants (L) was 0.35 ± 0.2 %ID/gram. A significant difference was found in the accumulation between the infected side (R) and the sterile side (L) after 13 days post-surgery of the ImmunoPET tracer (p = 0.003).

Short-term hematological effect assessment reveals minimal adverse effects

With the mean injected dose of 10.25 MBq of [^89^Zr]-4497 (n=9), no adverse effects were found on the white blood cell count, red blood cell count, or hemoglobin level for all animals in the [^89^Zr]-4497 (ImmunoPET) imaging groups including the excluded animal with an infected sterile side (left femur with implant) (Fig. S5). The median white blood cell count was 7.4 × 10^9^/L (IQR 5.6-14.0), normal range: 1.96-8.25 × 10^9^/L. The median of the Red blood cell count was 8.6 × 10^12^/L (IQR 8.24-10.1), normal range: 7.27-9.65 × 10^12^/L. The median of the hemoglobin level assessment was 13.2 g/dL (IQR 12.7-16.1), normal range: 13.7-17.6 g/dL. Lastly, thrombocytopenia was observed, showing a platelet count of 78 × 10^9^/L (IQR 14.5-171), normal range: 638-1177 × 10^9^/L.

**Supplementary table S1.** **Colony-forming-unit of the [^89^Zr]-4497 imaging group for sterile (S4.A) and infected (S4.B) leg**

| **Table S1,A** | ***Group*** | ***Sterile leg*** | | |  |
| --- | --- | --- | --- | --- | --- |
|  |  |  | **Surrounding bone** | **Articular capsule** | **Outcome** |
| *Animal 1* | [^89^Zr]-4497 (no co-injection) | *NDC* | *NDC* | *NDC* | Sterile |
| *Animal 2* |  | *NDC* | *NDC* | *NDC* | Sterile |
| *Animal 3* |  | *NDC* | *NDC* | *NDC* | Sterile |
| *Animal 1* | [^89^Zr]-4497 (300 µg co-injection) (10x) | *NDC* | *NDC* | *NDC* | Sterile |
| *Animal 2* |  | *NDC* | *NDC* | 3.5 x 10^3^ | Superficial infection |
| *Animal 3* |  | *NDC* | *NDC* | 2.9 x 10^5^ | Superficial infection |
| *Animal 1* | [^89^Zr]-4497 (600 µg co-injection) (20x) | *NDC* | *NDC* | 3.3 x 10^6^ | Superficial infection |
| *Animal 2* |  | *NDC* | *NDC* | 4.4 x 10^4^ | Superficial infection |
| *Animal 3* |  | 4.9 x 10^5^ | 2.5 x 10^5^ | 2.7 x 10^6^ | Contaminated (excluded) |

*NDC = no detectable colony-forming units*

| **Table S1,B** | ***Group*** | ***Infected leg*** | | |  |
| --- | --- | --- | --- | --- | --- |
|  |  | **Implant** | **Surrounding bone** | **Articular capsule** | **Outcome** |
| *Animal 1* | [^89^Zr]-4497 (no co-injection) | 4.0 x 10^6^ | 2.7 x 10^5^ | 3.2 x 10^5^ | Infection |
| *Animal 2* |  | 6.3 x 10^5^ | 4.0 x 10^6^ | 4.0 x 10^5^ | Infection |
| *Animal 3* |  | 5.0 x 10^5^ | 3.5 x 10^6^ | Lost sample | Infection |
| *Animal 1* | [^89^Zr]-4497 (300 µg co-injection) (10x) | 4.6 x 10^5^ | 4.9 x 10^5^ | Lost sample | Infection |
| *Animal 2* |  | 5.6 x 10^5^ | 1.2 x 10^6^ | 3.5 x 10^3^ | Infection |
| *Animal 3* |  | 1.4 x 10^5^ | 2.7 x 10^6^ | 2.9 x 10^5^ | Infection |
| *Animal 1* | [^89^Zr]-4497 (600 µg co-injection) (20x) | 4.9 x 10^5^ | 2.9 x 10^6^ | 5.2 x 10^6^ | Infection |
| *Animal 2* |  | 3.0 x 10^5^ | 1.9 x 10^6^ | 5.1 x 10^6^ | Infection |
| *Animal 3* |  | 5.3 x 10^5^ | 2.2 x 10^6^ | Lost sample | Infection (excluded) |

**Supplementary table S2. Colony-forming-unit of the conventional radiotracer imaging** **group (Sterile and Infected leg)**

| **Table S2,A** | ***group*** | ***Sterile leg*** | | |  |
| --- | --- | --- | --- | --- | --- |
|  |  | **Implant** | **Surrounding bone** | **Articular capsule** | **Outcome** |
| *Animal 1* | Radiotracers  [99mTc]Tc-MDP, [18F]FDG, and [18F]NaF | *NDC* | *NDC* | *NDC* | Sterile |
| *Animal 2* |  | *NDC* | *NDC* | *NDC* | Sterile |
| *Animal 3* |  | *NDC* | *NDC* | 2.9 x 10^3^ | Superficial infection |
| *Animal 4* |  | 2.7 x 10^5^ | 4.8 x 10^5^ | 3.8 x 10^6^ | Contaminated (excluded) |

*NDC = no detectable colony-forming units*

| **Table S2,B** | ***group*** | ***Infected leg*** | | |  |
| --- | --- | --- | --- | --- | --- |
|  |  | **Implant** | **Surrounding bone** | **Articular capsule** | **Outcome** |
| *Animal 1* | Radiotracers  [99mTc]Tc-MDP, [18F]FDG, and [18F]NaF | 1.7 x 10^6^ | 3.2 x 10^6^ | 2.0 x 10^6^ | Infection |
| *Animal 2* |  | 3.7 x 10^5^ | 1.7 x 10^6^ | 3.4 x 10^6^ | Infection |
| *Animal 3* |  | 3.5 x 10^5^ | 3.3 x 10^6^ | 3.5 x 10^7^ | Infection |
| *Animal 4* |  | 1.2 x 10^5^ | 5.2 x 10^6^ | 3.1 x 10^7^ | Infection (excluded) |

**Supplementary table S3. Mean uptake values (SUVbw) of the [^89^Zr]-4497 (ImmunoPET) imaging groups**

| ***[^89^Zr]-4497(no co-injection) (n=3)*** | | | | |  |
| --- | --- | --- | --- | --- | --- |
|  | Day +4 | Day +6 | Day +10 | Day +13 | |
| *Biofilm-infected side* | 4.11 | 4.28 | 4.15 | 5.64 | |
| *Sterile side* | 0.80 | 1.47 | 1.46 | 1.89 | |
| ***[^89^Zr]-4497 (300 µg co-injection cold 4497-antibody) (n=3)*** | | | | | |
|  | Day +4 | Day +6 | Day +10 | Day +13 | |
| *Biofilm-infected side* | 4.75 | 3.84 | 4.72 | 5.11 | |
| *Sterile side* | 1.88 | 1.23 | 1.96 | 2.48 | |
| ***[^89^Zr]-4497 (600 µg co-injection cold 4497-antibody) (n=3)*** | | | | | |
|  | Day +4 | Day +6 | Day +10 | Day +13 | |
| *Biofilm-infected side* | 4.94 | 4.34 | 2.80 | 5.51 | |
| *Sterile side* | 2.17 | 1.61 | 1.62 | 4.20 | |

**Supplementary table S4. Mean uptake values (SUVbw) of the radiotracer imaging group**

| ***Bone scintigraphy ([^99m^Tc]Tc-MDP SPECT/CT)*** | | | |
| --- | --- | --- | --- |
|  | Day +4  (n=3) | Day +10 (n=2) | Day +13 (n=2) |
| *Biofilm-infected side* | 4.67 | 5.08 | 4.69 |
| *Sterile side* | 4.31 | 4.23 | 4.47 |
| ***[^18^F]FDG PET/CT*** | | | |
|  | Day +4  (n=3) | Day +10 (n=2) | Day +13 (n=2) |
| *Biofilm-infected side* | 2.82 | 2.21 | 2.12 |
| *Sterile side* | 2.95 | 2.18 | 1.24 |
| ***[^18^F]NaF PET/CT*** | | | |
|  | Day +6  (n=2) | Day +12 (n=3) |  |
| *Biofilm-infected side* | 12.56 | 10.71 |  |
| *Sterile side* | 13.28 | 10.09 |  |

**References**

1. de Vor L, van Dijk B, van Kessel K, Kavanaugh JS, de Haas C, Aerts PC, et al. Human monoclonal antibodies against Staphylococcus aureus surface antigens recognize in vitro and in vivo biofilm. Elife. eLife Sciences Publications Ltd; 2022;11. https://doi.org/10.7554/eLife.67301

2. Allen KJH, Jiao R, Li J, Beckford-Vera DR, Dadachova E. In Vitro and In Vivo Characterization of 89Zirconium-Labeled Lintuzumab Molecule. Molecules. MDPI; 2022;27. https://doi.org/10.3390/molecules27196589

3. Lindmo T, Boven E, Cuttitta F, Fedorko J, Bunn PA. Determination of the Immunoreactive Fraction of Radiolabeled Monoclonal Antibodies by Linear Extrapolation to Binding at Infinite Antigen Excess 1. Journal oflmmunologicalMethods. 1984.

4. Fedorov A, Beichel R, Kalpathy-Cramer J, Finet J, Fillion-Robin J-C, Pujol S, et al. 3D Slicer as an image computing platform for the Quantitative Imaging Network. Magn Reson Imaging. 2012;30:1323–41. https://doi.org/10.1016/j.mri.2012.05.001

5. Giknis Mary Clifford Charles. Clinical Laboratory Parameters for CrL:WI (Han) [Internet]. Char. 2008 Mar. https://www.criver.com/sites/default/files/resources/rm_rm_r_Wistar_Han_clin_lab_parameters_08.pdf. Accessed 20 Sep 2024

**Figure Title and Legend**

Title: **Fig. S1 HPLC characterization shows radiopurity of Zirconium-89 labeled 4497-antibody with DFO conjugation.**

Legend: HPLC assessment was performed directly after radiolabeling on the day of administration **(a)** HPLC radio-trace of the [^89^Zr]-labeled 4497-antibody **(b)** HPLC UV-trace of [^89^Zr]-labeled 4497-antibody.

Title: **Fig. S2 Photographic clarification of the arthritis-induced thickened articular capsule.**

Legend: (**a**) In addition to the intrafemoral implant infection model, the joint itself became infected, leading to the development of an arthritis-induced thickened articular capsule. Based on the accumulation in the region superior to the knee joint, as observed with [^89^Zr]-4497 PET/CT analyses and confirmed through photographic evidence, the joint demonstrated the highest uptake of the ImmunoPET tracer. This finding was further validated by CFU assessment of the arthritis-induced thickened articular capsule. (**b**) Minimal to moderate thickening of the joint was observed on the sterile sides. Regardless of the thickness, the articular capsule was included in both the CFU assessment and *ex vivo* biodistribution analyses.

Title: **Fig. S3 Bacterial counts of implant, surrounding bone and articular capsule from the biofilm-infected side and sterile side validate sufficient infection induction and sterility.**

Legend: Colony-forming units per milliliter (CFU/mL) of implant, surrounding bone, and articular capsule from the (a) biofilm-infected implant side and (b) the sterile implant side of the ImmunoPET imaging groups. Four animals from both [^89^Zr]-4497 co-injections groups showed a contaminated articular capsule on the sterile side. (c) CFU/mL, of implant surrounding bone and articular capsule of the biofilm-infected implant side, and (d) the sterile implant side from the radiotracer imaging group. One animal showed a contaminated articular capsule on the sterile side.

Title: **Fig. S4 *Ex vivo* biodistribution assessment of the excluded animals displays sensitivity of the [^89^Zr]-4497 ImmunoPET tracer in finding a bacterial focus.**

Legend: Organs and implants were obtained and measured individually with the gamma-count machine at 13 days post-surgery. Tissue accumulation of the ImmunoPET tracer is presented in %ID/gram. Due to an infected “sterile” side. ImmunoPET accumulation was comparable between the infected and sterile side. The sterile side (femur with sterile implant and articular capsule) showed a combined %ID/gram of 2.61/ The biofilm-infected side showed a combined %ID/gram of 3.03.

Title: **Fig. S5 No adverse short-term effects on white blood cell count were observed in the animals during the 10-days follow-up period after a single administration of [^89^Zr]-4497 (ImmunoPET).** White blood cell count, red blood cell count, hemoglobin levels, and platelet counts were measured immediately post-termination. Dotted lines represent the normal for each corresponding hematological parameter. The median and range are reported. No significant decrease in WBC, RBC, or hemoglobin levels was observed following intervention with the ImmunoPET tracer. Thrombocytopenia was observed in all animals, likely due to the surgery on both femoral bones, Staphylococcus aureus bacteremia, and/or bone marrow infection.
